# Computer-assisted analysis of routine EEG to identify hidden biomarkers of epilepsy: A systematic review^☆^

**DOI:** 10.1016/j.csbj.2023.12.006

**Published:** 2023-12-10

**Authors:** Émile Lemoine, Joel Neves Briard, Bastien Rioux, Oumayma Gharbi, Renata Podbielski, Bénédicte Nauche, Denahin Toffa, Mark Keezer, Frédéric Lesage, Dang K. Nguyen, Elie Bou Assi

**Affiliations:** a Department of Neurosciences, University of Montreal, Canada; b Institute of biomedical engineering, Polytechnique Montreal, Canada; c University of Montreal Hospital Center’s Research Center, Canada; d Centre for Clinical Brain Sciences, University of Edinburgh, Edinburgh, United Kingdom; e School of Public Health, University of Montreal, Canada; f Stichting Epilepsie Instellingen Nederland (SEIN), Heemstede, the Netherlands

**Keywords:** Epilepsy, Electroencephalogram, Machine Learning, Diagnosis, Computer-assisted, Biomarker

## Abstract

**Background:**

Computational analysis of routine electroencephalogram (rEEG) could improve the accuracy of epilepsy diagnosis. We aim to systematically assess the diagnostic performances of computed biomarkers for epilepsy in individuals undergoing rEEG.

**Methods:**

We searched MEDLINE, EMBASE, EBM reviews, IEEE Explore and the grey literature for studies published between January 1961 and December 2022. We included studies reporting a computational method to diagnose epilepsy based on rEEG without relying on the identification of interictal epileptiform discharges or seizures. Diagnosis of epilepsy as per a treating physician was the reference standard. We assessed the risk of bias using an adapted QUADAS-2 tool.

**Results:**

We screened 10 166 studies, and 37 were included. The sample size ranged from 8 to 192 (mean=54). The computed biomarkers were based on linear (43%), non-linear (27%), connectivity (38%), and convolutional neural networks (10%) models. The risk of bias was high or unclear in all studies, more commonly from spectrum effect and data leakage. Diagnostic accuracy ranged between 64% and 100%. We observed high methodological heterogeneity, preventing pooling of accuracy measures.

**Conclusion:**

The current literature provides insufficient evidence to reliably assess the diagnostic yield of computational analysis of rEEG.

**Significance:**

We provide guidelines regarding patient selection, reference standard, algorithms, and performance validation.

## Highlights

• There is insufficient evidence to assess the diagnostic accuracy of computational analysis of routine EEG for epilepsy.
• Studies are at high risk of bias, mostly due to issues in patient selection and performance validation.
• We suggest guidelines for future studies on patient selection, reference standard, algorithms, and performance validation.

## Graphical Abstract

**Figure:**
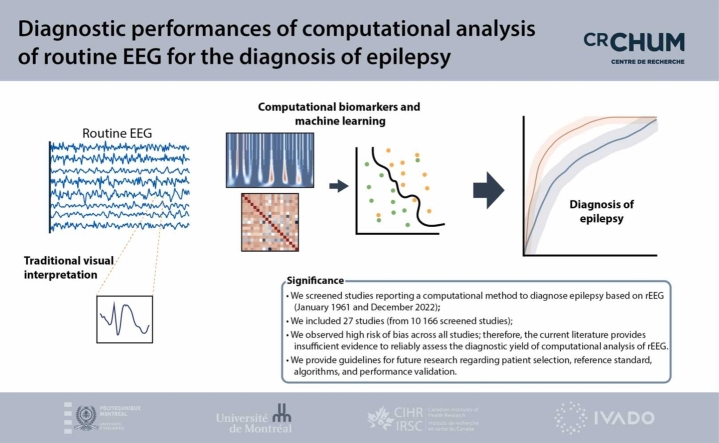

## 1 Introduction

Epilepsy is characterized by a chronic propensity towards epileptic seizures [1]. It is a common neurological condition, with an estimated period (lifetime) prevalence of 1% in the general population [2]. Diagnosing epilepsy poses a serious clinical challenge, with a ∼20% misdiagnosis rate [3], [4]. A false positive diagnosis can lead to unnecessary employment and lifestyle restrictions, adverse effects from medications, and social stigma, often for several years [5]. On the contrary, a delay in diagnosis and treatment can put the patient at risk for seizure-related injuries, road accidents, and death [6].

According to the International League Against Epilepsy (ILAE), the diagnosis of epilepsy requires at least two unprovoked epileptic seizures or a single unprovoked seizure with a risk of recurrence ≥ 60% over 10 years [1]. A short term (20- to 60-minute) scalp electroencephalogram (EEG), or routine EEG, can support a diagnosis after a first single unprovoked seizure. Interictal epileptiform discharges (IEDs) on routine EEG double the risk of recurrent seizures, thus allowing a diagnosis of epilepsy and generally warranting antiseizure medication (ASM) therapy [1], [7], [8].

While they are considered a hallmark of epilepsy, IEDs have limitations that impact the diagnostic utility of routine EEG for epilepsy. On the one hand, overinterpretation of EEG waveforms as IEDs can lead to an erroneous diagnosis of epilepsy [5]. Although the diagnosis of epilepsy is clinical and depends on a clear history of at least one unprovoked seizure [1], in reality, physicians often face an unreliable recounting of the suspected seizure event, and several paroxysmal disorders such as syncope can masquerade as seizures [9], [10]. In these situations, the moderate interrater reliability of IEDs (even among fellowship-trained neurophysiologists) can lead to epilepsy overdiagnosis [11], [12]. On the other hand, IEDs are elusive [13], [14]. In a systematic review of diagnostic accuracy studies assessing routine EEG after a first unprovoked seizure, the sensitivity of EEG was only 17% in adults [7]. Computer-assisted analysis has been proposed as an alternative to increase the test performance of EEG.

Several characteristics of brain activity on EEG may help identify people with epilepsy, including connectivity [15], [16], [17], signal predictability and complexity [18], [19], spectral power [20], [21], and chaoticity [22]. Discovering new, non-visible markers of epilepsy could increase the diagnostic yield of the EEG, improve its accessibility, and reduce costs, especially in settings where the expertise of a fellowship-trained neurophysiologist is unavailable [23], [24]. In spite of this, none of the proposed non-visible markers of epilepsy have translated into clinical practice [1], [8], [24], [25], [26]. Several narrative reviews have described potential biomarkers and EEG processing techniques [27], [28], [29], but there lacks a systematic review evaluating the population and methodological quality of these studies, and summarizing the diagnostic performance of these tools.

We performed a systematic review of diagnostic test accuracy of computational biomarkers (other than IEDs or electrographic seizures) extracted from routine EEG for the diagnosis of epilepsy.

## 2 Methods

We complied with our published protocol to conduct this study [30].

### 2.1 Study design

This study follows guidance from the Cochrane Diagnostic Test Accuracy group. We follow reporting standards set forth by the Preferred Reporting Items for Systematic Reviews and Meta-Analyses statement for diagnostic test accuracy (PRISMA-DTA) [31]. We considered studies in all languages published after 1961 (the first use of digital EEG [32]) up to the last review update (December 2022).

### 2.2 Study selection criteria

#### 2.2.1 Type of studies

We included retrospective or prospective diagnostic studies comparing at least one computed biomarker for the diagnosis of epilepsy on < 24 h scalp EEG (either in the inpatient or outpatient setting) between people with and without epilepsy that did not explicitly rely on the identification of IEDs or ictal activity (seizures). We excluded studies without human participants, studies that used long-term (>24 h), intracranial, or critical care recordings, studies that focused solely on seizure/spike detection or on short-term (<24 h) seizure prediction, as well as studies that did not include both individuals with and without epilepsy. For studies that included multiple EEG recoding settings (e.g., routine and critical care settings) and electrode location (e.g., both surface and intracranial), we only extracted data that met the inclusion criteria.

#### 2.2.2 Population

Our population of interest was individuals undergoing routine EEG in a clinical or research setting. We did not restrict the population to patients undergoing EEG after a first unprovoked seizure. Routine EEG was defined as a < 24 h scalp recording using the international 10–20 electrodes system, with or without prior sleep deprivation. There was no restriction on age, medication use, or co-morbidities.

#### 2.2.3 Reference standard

We defined the reference standard as the diagnosis of epilepsy, as determined by a physician, based on criteria specified by the study authors (clinical or para-clinical), so long as those criteria respected the definition of epilepsy by the International League Against Epilepsy (i.e., had at least one seizure and long-term enduring predisposition to other unprovoked seizures) [1], [33]. Alternative definitions (which do not rely on the presence of at least one seizure) were accepted for the qualitative analysis but excluded from meta-analyses.

#### 2.2.4 Index test

The index test is a characteristic or feature that is computationally extracted from the EEG signal to identify patients with epilepsy, without relying on the detection of IEDs or seizures. These include measures of connectivity, entropy, chaoticity, and power spectrum density [34], as well as statistical models that combine several features or models that directly use the raw EEG signal as their input. We included studies that computed the biomarkers from the same EEG used to diagnose epilepsy, although this was considered in the evaluation of the risk of bias (see **Risk of bias**).

### 2.3 Search strategy

The search strategy (**Appendix 1**) was developed by two medical librarians specialized in knowledge synthesis (BN and RP). We searched MEDLINE (Ovid), EMBASE (Ovid), EBM reviews (Ovid), IEEE Explore along with grey literature (see **Appendix 1** for details) for articles, conference papers and conference abstracts published between December 1961 and December 2022. We used the Covidence platform (Melbourne, Australia) to manage study selection and data collection. Two independent, mutually blinded reviewers (EL, and either JNB or BR) screened the records for eligibility by title and abstract. Any item deemed relevant by any reviewer was independently assessed for final inclusion from its full text by the same reviewers. Conflicts regarding inclusion were resolved by consensus.

### 2.4 Data collection

Two independent reviewers (EL and OG) extracted pre-specified data while blinded to the verdict of the other reviewer using a custom extraction form tested on the first five articles. Any conflicting data were re-assessed and resolved by consensus. Corresponding authors were contacted through their electronic address if data of interest were not available in the original publication. Data collection included the following information: 1) Title, authors, country of sampling, year of publication; 2) Study type (retrospective vs. prospective, design); 3) Study sample (inclusion/exclusion criteria, number of screened/included patients); 4) Data collection (number of patients and EEGs, duration of EEGs, recording protocol, participants characteristics); 5) Reference standard (definition, application to all patients, time-lapse with EEG); 6) Index test (preprocessing, segment selection, feature extraction and selection, classification algorithm and methodology, reporting of performance); and 7) Measurements of diagnostic test validity (e.g., accuracy, sensitivity, specificity). These items are further detailed in the pre-published protocol [30].

### 2.5 Study reproducibility

Two independent reviewers (EL and OG) assessed study reproducibility. A study was judged reproducible when, given access to the data, the processing methodology and machine learning (ML) methods were sufficiently detailed such that the experiment could be fully reproduced. More specifically, the following items were assessed: objective criteria for selection of EEG segments, code and data availability, and reporting of key methodological details (preprocessing [filtering, channel selection, artifact detection and removal, segmentation], ML optimization [feature extraction and selection, choice of ML model, hyperparameter tuning], and ML evaluation).

### 2.6 Risk of bias

The risk of bias of all included studies was assessed through a version of the QUADAS-2 tool adapted for the characteristics of this review [30], [35]. Two independent and mutually blinded reviewers (EL and OG) assessed the risk of bias for each of the following four elements as low, high, or unclear: 1) Patient selection (representativeness of clinical practice, identical inclusion/exclusion criteria for all participants, exclusion of individual EEG/EEG segments); 2) Index test (identical EEG protocols for all patients, validation of the index test on an independent sample); 3) Reference standard (specified criteria for the diagnosis of epilepsy, independence of the diagnosis to the index test); and 4) Flow and timing (whether the whole sample underwent the same reference standard, timing between index test and epilepsy diagnosis, exclusion of EEG or EEG segments during the evaluation). Any conflicting interpretations were resolved by consensus. These criteria are further detailed in the pre-published protocol [30].

### 2.7 Data synthesis

We planned to report the pooled sensitivity and specificity estimates for studies providing the number of true/false positives/negatives, and the area under the receiver operating characteristic curve (AUROC) for studies that provided a varying threshold. We planned a meta-analysis of diagnostic performances, a quantitative assessment of heterogeneity, and subgroup analyses [30]. However, due to excessive methodological heterogeneity among included studies, we concluded a meta-analysis would not help interpret our results and decided to report a qualitative assessment only (see **Results: Risk of bias and applicability**).

### 2.8 Quality of evidence

The quality of evidence for the primary outcome was evaluated by two authors (EL and OM) based on the GRADE criteria for diagnostic test accuracy, [36] recognizing that the GRADE approach is designed for pooled estimates. Data from cross-sectional or cohort study which included patients with diagnostic uncertainty for epilepsy started at “high quality”, while data from other observational designs started at “low quality”. We downgraded the evidence by one level for high risk of bias, indirectness, inconsistency, imprecision, and high probability of publication bias, and we upgraded the quality by one level for large effect size.

## 3 Results

### 3.1 Study selection

**Fig. 1:**
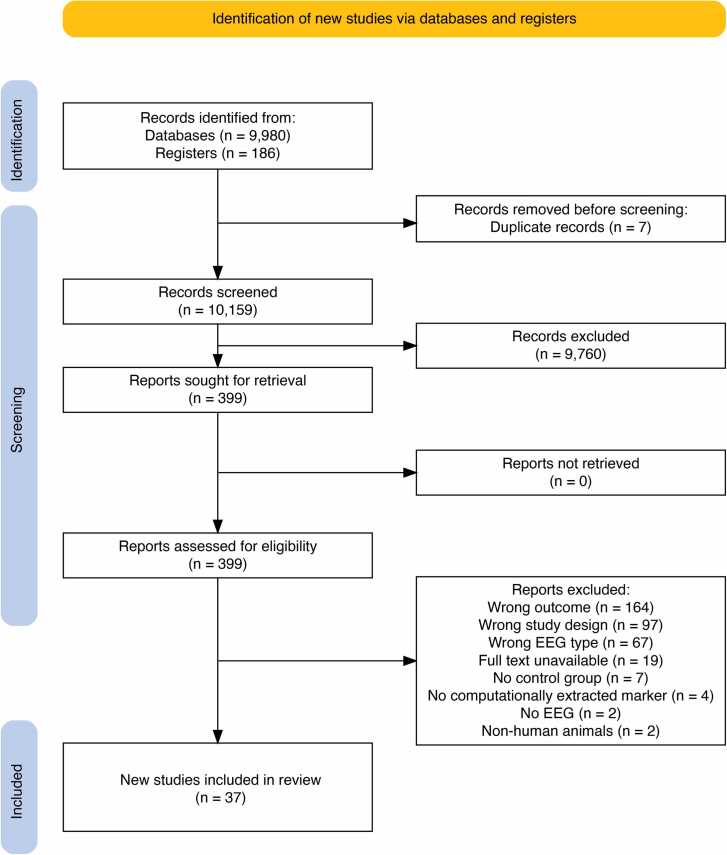
PRISMA flowchart of the study selection, screening, and assessment.

The study selection flow diagram is presented in Fig. 1. Our initial search yielded 10 166 items. After removal of duplicates, title and abstract screening, and full text review, we included 37 studies. The most common reasons for exclusion pertained to study outcome (e.g., seizure or interictal spike detection) in 164 studies (45% of final exclusions), study design (e.g., no diagnostic accuracy testing) in 97 studies (27%), and EEG type (e.g., intracranial, critical care, or long-term monitoring) in 67 studies (19%).

### 3.2 Study characteristics

**Fig. 2:**
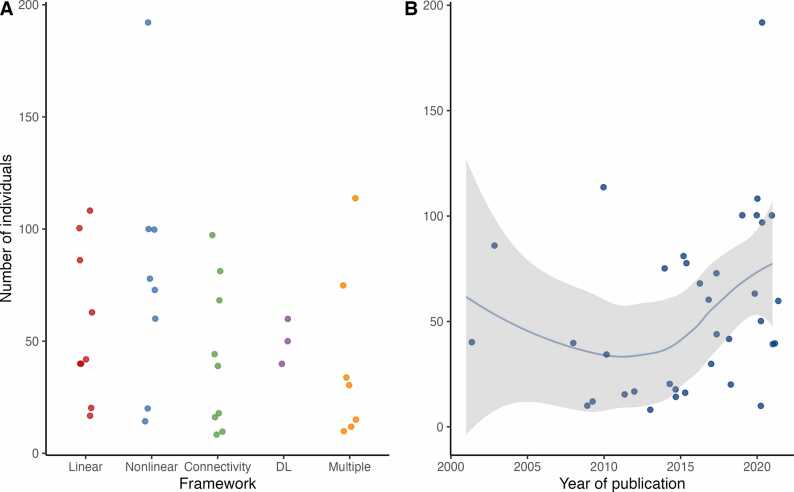
Sample size of included studies. A: Number of individuals included in the assessment of computational biomarkers per study. B: Sample size of included studies by year of publication, with a moving average and 95% standard error overlay. Studies with unclear number of participants are not shown.

We describe included studies in Table 1. The sample size ranged from 8 to 192 (mean=54.4; Fig. 2), while only six studies (16%) included ≥ 100 subjects [37], [38], [39], [40], [41], [42]. Years of publication ranged from 2001 to 2022; twelve studies (32%) were published after 2020. Most studies included both children (i.e., aged <=18 years old; n = 18; 49%) and adults, whereas 11 studies (30%) only included children [18], [43], [44], [45], [46], [47], [48], [49], [50], [51], [52] and eight (22%) only included adults [37], [38], [39], [42], [53], [54], [55], [56]. Twenty-four studies (65%) included any type of epilepsy, whereas seven studies (19%) only included generalized epilepsy [15], [18], [45], [53], [57], [58], [59] and six (16%) only included focal epilepsy [39], [54], [55], [56], [60], [61]. Type of epilepsy, however, was not available in thirteen studies (35%). Five studies (14%) only included patients with electro-clinical syndromes (absence epilepsy [45], idiopathic generalized epilepsies [15], [57], [59], epileptic encephalopathy with spike-wave activation in sleep [50]).

**Table 1:** Characteristics of included studies.

**Study**	**Country**	**Epilepsy type**	**Total sample size**	**Group**	**Description**	**Age range**	**Sex (F/M)**	**Comorbidities**	**Number of ASM**	**Framework**
Cao, 2021	UK	Generalized	39	Epilepsy	15 PWE	33 ± 12	10/5	None	0–2	Connectivity
				No epilepsy	10 HC and 14 with NEAD	HC: 37 ± 15 NEAD: 33 ± 13	HC: 6/4 NEAD: 10/4	NEAD (14)	HC: 0 NEAD: 0–4	
Guerrero, 2021	Colombia	NA	40	Epilepsy	20 PWE (TUH Epilepsy corpus)	NA	NA	NA	NA	DL, Linear
				No epilepsy	20 w/o epilepsy who underwent a rEEG (TUH Epilepsy corpus)	NA	NA	NA	NA	
Rijnders, 2021	United States	NA	60	Epilepsy	30 PWE (TUH Epilepsy corpus)	52.5 (mean)	19/11	Stroke (3), DM (2), dementia, HBV/HCV (NA)	NA	DL, connectivity
				No epilepsy	20 w/o epilepsy who underwent a rEEG (TUH Epilepsy corpus)	53.7 (mean)	17/13	Stroke (8), DM (3), dementia (2), HBV/HCV (2)	NA	
Zelig, 2021	Israel	Focal, generalized, unknown	100	Epilepsy	28 admitted to the ED after first seizure who developed epilepsy	51.4 ± 20.9	12/16	Headache, brain tumors, IC hemorrhage, MG, depression, AD/HD, autism, schizophrenia, anxiety, substance abuse.	Unclear	Linear
				No epilepsy	42 admitted to ED after fst sz who remained seizure-free & 30 patients undergoing rEEG for neuropsychiatric diseases	Fst sz: 48.5 ± 17.8 Others: 55.1 ± 3.1	Fst sz: 15/27 Others: NA	Similar to cases for fst sz patients; NA for others	Unclear	
Ahmadi, 2020	Belgium	NA	10	Epilepsy	5 PWE	NA	NA	NA	NA	Connectivity, nonlinear
				No epilepsy	5 with PNES	NA	NA	PNES	NA	
Lin, 2020	Taiwan	Focal and generalized	50	Epilepsy	25 PWE	4–17	9/16	NA	NA	DL
				No epilepsy	25 with Tourette’s syndrome or syncope	4–15	NA	Tourette’s syndrome (92%), syncope (8%)	NA	
Ouyang, 2020	Taiwan	Generalized epilepsy	63	Epilepsy	23 with GE	5–18	10/13	0	0–1	Linear
				No epilepsy	23 age-matched HC	5–18	21/19	NA	0	
Prahbu, 2020	Guinea-Bissau	NA	97	Epilepsy	51 PWE	12–38	21/30	NA	NA	Connectivity
				No epilepsy	46 HC	17–33	5/41	NA	NA	
Song, 2020	China	NA	100	Epilepsy	50 PWE	29.59 ± 4.34	25/25	NA	NA	Nonlinear
				No epilepsy	50 age-matched HC	26.86 ± 3.69	25/25	NA	NA	
Uyttenhove, 2020	Belgium	NA	NA	Epilepsy	PWE (TUH Epilepsy corpus)	NA	NA	NA	NA	DL
				No epilepsy	Patients w/o epilepsy who underwent a rEEG (TUH Epilepsy corpus)	NA	NA	NA	NA	
Varatharajah, 2020	United States	Focal	192	Epilepsy	48 with DRFE	18–66	25/23	NA	NA	Nonlinear
				No epilepsy	144 HC	20–77 (before exclusion)	82/121 (before exclusion)	NA	NA	
Yağmur, 2020	Turkey	NA	108	Epilepsy	88 PWE	NA	NA	NA	NA	Linear
				No epilepsy	20 HC	NA	NA	NA	NA	
Panwar, 2019	India	Focal, generalized, focal and generalized	100	Epilepsy	50 PWE (gen., focal, and LGS)	6–69	16/34	NA	NA	Nonlinear
				No epilepsy	50 HC	6–79	20/30	NA	NA	
Tripathi, 2018	India	NA	20	Epilepsy	10 PWE	3–5	3/7	NA	NA	Linear
				No epilepsy	10 HC	3–5	3/7	NA	NA	
V, 2018	India	Focal	42	Epilepsy	21 with TLE	19–31	0/21	NA	2.66 (mean)	Linear
				No epilepsy	21 HC from existing imaging data bank	24–32	0/21	NA	NA	
Bosl, 2017	United States	Generalized	73	Epilepsy	26 with absence seizures	8.6 (1.7)	13/13	NA	NA	Nonlinear
				No epilepsy	47 undergoing rEEG w/o epilepsy	7.74 (4.3)	15/9	ASD	NA	
Mazzucchi, 2017	Italy	Focal	44	Epilepsy	22 with cryptogenic FE	18–76	13/9	NA	0–4	Connectivity
				No epilepsy	22 age-matched HC	20–73	6/16	NA	NA	
Tibdewal, 2017	India	Focal, generalized	60	Epilepsy	30 with DRFE undergoing pre-surgical evaluation	NA	NA	NA	NA	Nonlinear
				No epilepsy	30 HC	NA	NA	NA	NA	
Uriguen, 2017	Spain	Generalized	30	Epilepsy	20 with IGE	11–70	14/6	NA	0–3	Linear, nonlinear
				No epilepsy	10 HC	23–60	3/7	NA	NA	
Schmidt, 2016	UK	Generalized	68	Epilepsy	30 patients with IGE w/o ASM	NA	NA	NA	0	Connectivity
				No epilepsy	38 HC	NA	NA	NA	NA	
Dasgupta, 2015	India	Generalized	81	Epilepsy	51 with GE	F: 15.21 (mean), M: 13.46 (mean)	26/25	NA	NA	Connectivity
				No epilepsy	30 HC	F: 16.87 (mean), M: 17.67 (mean)	15/15	NA	NA	
Pyrzowski, 2015	Poland	Focal	78	Epilepsy	51 with TLE or FLE, mostly hospitalized for ASM resistance	18–68	36/15	Mood disorder (4), cardiac disease (6), neurosis (2), stroke (2), cerebral palsy, cognitive impairment (2), brain tumor	0 (4), 1 (12), 2 (20), 3 (15)	Nonlinear
				No epilepsy	13 with vEEG confirmed PNES & 14 admitted for headaches	19–57	22/5	Mood disorder (2), migraine (2), meningitis, opioid usage disorder	0 (14), 1 (12), 2 (1)	
Rajaei, 2015*	United States	Focal, generalized	14	Epilepsy	7 PWE	2–14	¾	NA	NA	Nonlinear
				No epilepsy	7 HC	8–18	¾	NA	NA	
Sargolzai, 2015 (1)*	United States	Focal, generalized	16	Epilepsy	9 PWE	4–15	4/5	NA	0	Connectivity
				No epilepsy	7 HC	8–18	¾	NA	0	
Sargolzai, 2015 (2)	United States	Focal, generalized	18	Epilepsy	11 PWE	8–18	5/6	NA	NA	Connectivity
				No epilepsy	7 HC	2–15	3/4	NA	NA	
Schmidt, 2014^***^	UK	Generalized	75	Epilepsy	35 with IGE	18–59	21/14	NA	0–4	Connectivity, linear
				No epilepsy	40 HC	30.7 (mean)	20/20	NA	NA	
Yang, 2014	China	NA	20	Epilepsy	10 with ESES	3–9	6/4	NA	NA	Nonlinear
				No epilepsy	10 HC	3–9	6/4	NA	NA	
Sargolzaei, 2013*	United States	Focal, generalized	8	Epilepsy	4 PWE	NA	2/2	NA	NA	connectivity
				No epilepsy	4 HC	NA	2/2	NA	NA	
Cabrerizo, 2012*	United States	Focal, generalized	17	Epilepsy	9 PWE undergoing rEEG	1–15	3/6	NA	0	Linear
				No epilepsy	8 patients w/o epilepsy undergoing rEEG	8–18	3/5	NA	0	
Chaovalitwongse, 2011^**^	United States	NA	15	Epilepsy	10 PWE undergoing rEEG	NA	NA	NA	NA	Linear, connectivity
				No epilepsy	5 patients undergoing rEEG	NA	NA	NA	NA	
Douw, 2010	Netherlands	Focal, generalized	114	Epilepsy	57 PWE who underwent routine EEG after a first seizure	50 (SD: 18)	29/28	White matter abnormalities, brain tumor, cortical atrophy, arachnoid cyst	0–1	Connectivity, linear
				No epilepsy	57 age-matched patients w/o epilepsy who underwent routine EEG after a fst sz	54 (17)	29/28	Stress, syncope, TIA, brain contusion, neuropathy, sleeping disorders, hypoglycemia, migraine, drug abuse, motor neuron disease, orthostatic hypotension, white matter abnormalities, brain tumor, cortical atrophy.	0	
Luo, 2010	China	NA	34	Epilepsy	21 PWE	NA	NA	NA	NA	Linear, nonlinear
				No epilepsy	13 HC	NA	NA	NA	NA	
Bao, 2009	China	NA	12	Epilepsy	6 PWE	NA	NA	NA	NA	Linear, nonlinear
				No epilepsy	6 HC	NA	NA	NA	NA	
Fan, 2009^**^	United States	Focal	10	Epilepsy	5 DRTLE	NA	NA	NA	NA	Connectivity
				No epilepsy	5 HC	NA	NA	NA	NA	
Cassar, 2008	Greece	Focal, generalized	40	Epilepsy	20 PWE	9–13	11/9	None	NA	Linear
				No epilepsy	20 age- and sex-matched HC	9–13	11/9	None	NA	
Poulos, 2003	Greece	NA	86	Epilepsy	42 PWE	NA	NA	NA	NA	Linear
				No epilepsy	44 with non-epileptic loss of consciousness	NA	NA	NA	NA	
Ruseckaite, 2001	Lithuania	Focal	40	Epilepsy	PWE	NA	NA	NA	NA	Linear
				No epilepsy	HC and head trauma patients	NA	NA	NA	NA	

* Same patients as ref.^49^^**^Same patients as ref.^62^^***^Same patients as ref.^15^ ASM: antiseizure medication; Db: diabetes; DL: deep learning; DR: drug-resistant; ESES: electrical status epilepticus during slow-wave sleep; FE: focal epilepsy, FLE: frontal lobe epilepsy; GE: generalized epilepsy; HBV: hepatitis B virus; HC: healthy controls; HCV: hepatitis C virus; IED: interictal epileptiform discharge; IGE: idiopathic generalized epilepsy; NA: Not available; NEAD: non-epileptic attacks disorder; PNES: psychogenic non-epileptic seizures; PWE: patients with epilepsy; rEEG: routine electroencephalography; TLE: temporal lobe epilepsy; vEEG: video-electroencephalography.

Thirteen studies (35%) provided a definition for the reference standard (diagnosis of epilepsy) [15], [18], [37], [38], [41], [42], [45], [52], [53], [62], [63], [64], [65]. In seven studies (19%), the diagnosis was based on a history of two or more seizures, or one seizure with abnormal neuroimaging or IED on EEG [18], [37], [41], [42], [43], [52], [53]. Three studies (8%) based the diagnosis of epilepsy on EEG features only [15], [45], [62], and three based the diagnosis on the EEG report mentioning a diagnosis of epilepsy [63], [64], [65]. The index tests are described in the section Signal processing and machine learning, and the computational biomarkers that were used are listed in Table 2.

**Table 2:** EEG recording and pre-processing details for each study.

**Study**	**EEG duration**	**Electrodes (N)**	**Sampling freq. (Hz)**	**Automated artifact detection**	**Frequency bands**	**Manual segment selection**	**Criteria for segment selection**	**Segment duration (s)**	**Overlapping segments**	**Montage**	**Channel selection**	**Criteria for channel selection**
**Cao, 2021**	72 s for HC, 48 s for EG and NEAD	21	500	None	0.79–4, 4–8, 8–15,15–32, > 32	Yes	No interictal abnormalities and relatively artifact-free	4	No	Bipolar	Manual	Removed Fp1 and Fp2 due to high levels of eye blink artifacts
**Guerrero, 2021**	20–30 min	21	250, 256, 400, 512 Hz	NA	NA	NA	NA	NA	NA	Bipolar (longitudinal)	None	-
**Rijnders, 2021**	20 min	21	250	ICA (removed component with highest correlation with Fp1) and trend line removal	1–4, 5–7, 8–13, 14–29, 30–55	Yes	Calmest segment	50	No	Referential (avg)	None	-
**Zelig, 2021**	20 min	19	512	NA	1–4, 4–8, 8–12, 12–20, 20–30, 30–40	No		Entire recording	-	NA	None	-
**Ahmadi, 2020**	3 h	27	256	None	1–4, 4–8, 9–13, 13–30, 30–40	Yes	IED-free, least amount of noise or artifacts	16	No	Referential (G2)	None	-
**Lin, 2020**	20	19	256	None	0.5–60	Yes	No eye movement or muscle artifacts, no segments from IPS nor HV	2	0%, 50%, 90%, 95%,	Referential (Cz)	None	-
**Ouyang, 2020**	20 min	19	256	None	0.5–60	Yes	Artifact-free	5	No	Referential (Cz,)	None	-
**Prahbu, 2020**	5	14	128	NA	NA	NA	NA	NA	NA	NA	Automated	Best performing subset in classification task
**Song, 2020**	2 min	16	512	ICA (NA)	1–4, 4–8, 8–13, 13–30	Yes,	No obvious signal loss	20	No	NA	None	-
**Uyttenhove, 2020**	NA	19	256	None	0.5–128	No		10	No	Referential (avg)	None	-
**Varatharajah, 2020**	16 min (controls), NA (cases)	62 (controls), 31 (cases)	2500 Hz (controls), 256 Hz (cases)	ICA (manual selection)	7.5–10.5 10.5–13.5	Yes	Controls: segments with artifacts. Cases: segments with eye closure and no epileptiform activity	10	No	Bipolar	Manual	Artefactual channels (4)
**Yağmur, 2020**	18 min	16	200	ICA (NA)	0.1–500	NA	NA	NA	NA	NA	None	-
**Panwar, 2019**	5 min	17	250, 256	None	0.5–15	Unclear	Unclear	1	Yes	Referential (avg)	None	-
**Tripathi, 2018**	30	19	NA	NA	1–4, 4–8, 8–13, 13–30	NA		NA	NA	Referential	Manual	NA
**V, 2018**	NA	32	5000	Average subtraction method considering R peaks as reference[135] and ICA (maual selection)	2–20	Yes	First 120 s artifact-free segment	120	No	Referential (avg)	None	-
**Bosl, 2017**	30 s (Hospital subjects) 12 s (Laboratory subjects)	19	200 Hz (Hospital subjects) 500 Hz (Laboratory subjects)	None and NetStation software artifact detection tool (ASD group, manual selection of artifactual components)	None, 0.1–100	Yes, Unclear	Visual review to select 30-s samples containing no spikes or evidence of epileptiform activity and with no artifacts, Exclude segments with eyes saccades and blinks. (automatically detected artifacts)	30, 12	No, No	Average, Average	None and manual	19 channels are selected corresponding to the electrode locations for hospital patients
**Mazzucchi, 2017**	15 min	19	128	None	1–4, 5–7, 8–13, 14–30, 31–60	Yes	Absence of artifacts, absence of IEDs	2 s	NA	NA	None	-
**Tibdewal, 2017**	12–15 min	19	114	NA	NA	NA	NA	8	NA	NA	Manual	Removed O1-O2 (corrupted data during acquisition)
**Uriguen, 2017**	NA	32	200	Kurtosis threshold or statistical outliers (threshold: 3 SD)	0.5–70	Yes,	No seizure activity, no epileptiform patterns	10.24	No,	Referential (mastoids)	None	-
**Schmidt, 2016**	NA	19	256	None	6–9, 8–13	Yes	Artifact free and GSW free	20	No	NA	None	-
**Dasgupta, 2015**	20–30 min	16	NA	ICA + neural network (underspecified) with manual selection of ICs	4–60	Yes	Noise-free	NA	NA	NA	None	-
**Pyrzowski, 2015**	20 min (19.5–22.1)	19	250	None	4–13	NA	NA	1–120	NA	Referential	None	-
**Rajaei, 2015**	NA	19	200, 512	None	0.5–70	Yes	Free of artifacts and ictal events	10	No	Referential	None	-
**Sargolzai, 2015 (1)**	NA	19	200, 500, 512	ICA[136] (NA)	NA	Yes	No seizures and no artifacts	9–90	NA	Referential (avg)	None	-
**Sargolzai, 2015 (2)**	NA	19	200, 500, 512	None,	NA,	Yes	Artifact-free and seizure-free	9	50%,	Referential	None	-
**Schmidt, 2014**	50	19	256	None	1–3, 3–6, 6–9, 10–14, 15–30, 30–70	Yes	Artifact-free, eyes closed	20	No	Referential	None	-
**Yang, 2014**	NA	16	500	None	0.5–35	Yes	No artifacts	8	NA	NA	None	-
**Sargolzaei, 2013**	NA	19	NA	None	0.1–70	NA	NA	NA	NA	Referential	None	-
**Cabrerizo, 2012**	20–40 min	19	500 and 512	None	< 4, 4–8, 8–13, 13–20, 20–36, 36–44	Yes	Free of artifacts, free of seizures, eyes closed	1	No	Referential	No	-
**Chaovalitwongse, 2011**	13–45 min	14–18	200 and 250	None	NA	No	Random sampling	60, 120, 180, 240	NA	Bipolar,	Manual	Channels that were consistent across EEGs
**Douw, 2010**	30 min	21	500	None	0.5–4, 4–8, 8–10, 10–13, 13–30, 30–45, 55–80	Yes	Artifact-free segments	8	No	Referential average	Manual	Fp1–2 and A1–2
**Luo, 2010**	NA	NA	NA	NA	NA	NA	NA	NA	NA	NA	NA	-
**Bao, 2009**	NA	22	200	None	2–34 (1 Hz incr.), 2–34 in (2 Hz incr.), 2–34.5 in (2.5 Hz incr.)	No		20.48, 40.96	NA	Referential, NA	None	-
**Fan, 2009**	20–30 min	19	250	UNICA[137]	NA	No	Random sampling	30	NA	Referential	None	-
**Cassar, 2008**	NA	30	400	None	0–4, 4–8, 8–13, 13–30, 30–45, 45–90	Yes	Free of technical and biogenic artifacts	10.24	No	Referential (A1 + A2)	None	-
**Poulos, 2003**	20 s	2	200	None	5–70	Yes	No epileptiform discharges	20	Yes	O2-Cz	Manual	Channel with “best” PDR
**Ruseckaite, 2001**	45 s	16	NA	NA	NA	NA	NA	3	No	NA	None	-

EG: Epilepsy group; HC: Healthy controls; ICA: Independent component analysis; NEAD: Non-epileptic attack disorder; PDR: Posterior dominant rhythm.

Three public datasets were used by five of the included studies (14%). Three studies used the Temple University Hospital (TUH) EEG dataset (“Epilepsy corpus”), with different sets of inclusion and exclusion criteria, resulting in sample sizes of 40–60 patients (for one study, the final sample size was not available) [63], [64], [65]. One study used the Emotiv dataset, a case-control dataset with 97 subjects recorded with an Emotiv low-cost scalp EEG helmet [66]. One study used the LEMON EEG dataset for the control group only [39].

### 3.3 Risk of bias and applicability

**Fig. 3:**
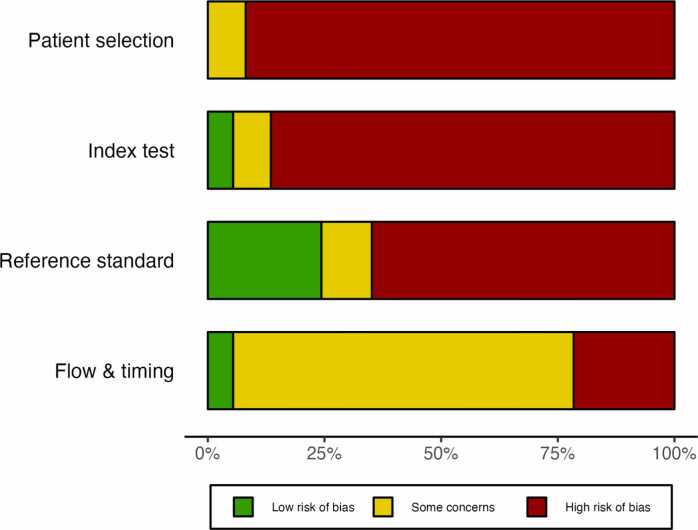
Summary of the risk of bias for each of the PRISMA domains.

Risk of bias was high or unclear in at least two domains for all studies (Fig. 3). The final consensus for each study and the description of the assessments are provided as supplementary materials (Fig. S1 **and** Table S2). For patient selection, no study had a low risk of bias. The most common reason for a high risk of bias in this domain was the use of distinct inclusion and exclusion criteria for subjects with and without epilepsy (e.g., patients with a diagnosis of epilepsy undergoing presurgical evaluation for cases, and healthy individuals for controls). Other reasons were the exclusion of patients without proper justification, and a study population that was not representative of clinical practice. For the index test, two studies had a low risk of bias [43], [65]. High risk of bias in this domain was frequently attributed to failure to validate the index test on an independent sample of patients. In four cases (11%), the EEG recording protocol or setting was different for cases and controls [15], [39], [53], [62]. For the reference standard domain, nine studies (24%) had a low risk of bias [15], [18], [37], [38], [41], [42], [45], [52], [53]. A common reason for a high risk of bias included failure to provide a definition for the reference standard. Finally, for the flow and timing domain, two studies had a low risk of bias [62], [65]. For most studies, the risk of bias was unclear because of an unspecified reference standard. Eight studies (22%) had a high risk of bias in this last domain because they used a different reference standard for cases and controls.

### 3.4 Results of individual studies

Reports of performances for individual studies must be interpreted in the context of high risk of bias in several domains. Diagnostic performances are reported in Table 3. The diagnostic accuracy ranged from 64% to 100%. Three studies (8%) provided a measure of statistical precision on their diagnostic performance metrics [41], [48], [55]. In the absence of pooled estimates, we assessed applicable GRADE criteria. The evidence quality was judged very low, starting at “low” for the study design and downgraded for high risk of bias, inconsistency (high variability in reported accuracy), and indirectness of evidence (differences between the studied and target populations). Publication bias and imprecision were omitted, as only three studies reported statistical precision.

**Table 3:** Biomarkers assessed in included studies by computational framework.

**Framework**	**Feature**	**Studies**
Linear	Power spectral density	[39], [44], [52], [63], [65], [67]
	Peak alpha frequency	[15]
	Mode of frequency spectrum	[61]
	Prediction error of autoregressive model	[18]
	Auto-correlation coefficient or standard deviation	[69], [70]
	Hjorth parameters (activity, mobility, complexity)	[51], [67]
	Statistical features (average, variance, standard deviation, skewness, kurtosis, Euclidean distance, T-Statistical distance, interquartile range, mutual information)	[40], [62], [72]
	Paroxysmal slow wave events (rate per min)	[37]
Nonlinear	Shannon entropy	[68], [70]
	Spectral entropy	[57], [68]
	Approximate entropy	[66], [70]
	Permutation entropy	[50]
	Multiscale entropy	[45], [50]
	Fuzzy entropy	[72]
	Renyi entropy	[68]
	Fractal dimension	[67], [68]
	Hurst indices	[70]
	Zero-crossings interval analysis	[56]
	Recurrent quantitative analysis	[45]
	Characteristic response analysis	[41]
	Bispectrum magnitude (average and variance)	[72]
	Periodicity	[70]
	Kolmogorov complexity	[66]
Connectivity	**Connectivity measures**	
	Mutual information	[53]
	Coherence	[53]
	Lagged coherence	[55]
	Phase-locking value	[15], [53]
	Pearson’s correlation coefficient	[53], [58]
	Euclidean distance	[60], [62]
	Cosine similarity	[46], [47], [48], [49]
	Horizontal visibility graph	[68]
	Synchronization likelihood	[42]
	Granger causality	[64]
	Phase-space recurrence	[46]
	Tucker decomposition	[38]
	Transfer entropy	[38]
	**Connectivity features**	
	Statistical (maximum, mean)	[53]
	Average degree	[15], [46], [47], [48]
	Closeness or betweenness centrality	[47], [48], [49], [68]
	Density	[46], [47], [48], [49], [58]
	Energy	[47], [48], [49], [58]
	Clustering coefficient	[38], [47], [48], [49], [55], [58], [68]
	Network efficiency	[38], [58]
	Rich club coefficient	[46], [47], [48], [49], [58]
	Small world index	[58]
	S-metric	[46], [47], [48], [49]
	Characteristic path length	[48], [55]
	Average vertex eccentricity	[48]
	Graph radius	[46], [48]
	Largest eigenvalue	[68]
	**Other connectivity-based features**	
	Dynamical connectivity analysis (local and critical coupling constant, global order parameter)	[15], [59]
	Microstates analysis (occurrence, duration, time coverage)	[54], [68]
Deep learning	No feature extraction	[43], [65]
	Prior feature extraction	[63], [64]

We analysed how performance was impacted by study size and risk of bias (Fig. S2). Sample size did not correlate with diagnostic performance. There was no clear trend towards inflated performances for studies at high risk of bias in any of the QUADAS-2 domains although no study had low risk of bias for the Patient selection domain. The inter-test variability was smaller for AUROC than for accuracy. There was a visible trend towards reduced inter-test variability among studies with low risk-of-bias in the Index test (Accuracy and AUROC), Reference standard (AUROC only), and Flow and timing (AUROC only) domains.

## 4 EEG processing and machine learning methods

EEG processing methods for each study are described in Table 2. Some technical terms related to EEG processing and machine learning are further defined in Table 4.

**Table 4:** Performance of computational EEG biomarkers for the diagnosis of epilepsy.

**Study**	**Classifier; feature (s)**	**Sens (%)**	**Spec (%)**	**Acc (%)**	**Prec (%)**	**Rec (%)**	**F**_**1**_**(%)**	**AUROC**	**Data leakage**	**Statistical testing**
**Cao, 2021**	kNN; CohMean beta, eyes closed (F4C4-FzCz): Epi vs HC			97.22				0.983	Yes	No
	kNN; CohMean beta, eye closed, (F3C3-FzCz): Epi vs HC							0.969	Yes	No
	kNN; CohMean beta, eye closed, (CzPz-C4P4): Epi vs HC							0.888	Yes	No
	kNN; CohMean beta, eye closed, (C3Cz-P3Pz): Epi vs HC							0.929	Yes	No
	kNN; MI delta, eye open, (T4T6-P4Pz): Epi vs NEAD			74.44					Yes	No
	kNN; PLV gamma, eye open (T3C3-CzPz): Epi vs NEAD			74.24					Yes	No
**Guerrero, 2021**	LR; relative band power (best model)			73.3	73.9	68	70.8	0.71	Yes	No
	ANN; relative band power (best model)			86.1	81	84	82.4	0.95	NA	No
	SVM; relative band power (best model)			77.3	77.5	74.3	75.8	0.78	NA	No
	CNN; relative band power (best model)			61.5	62.2	58.7	60.4	0.60	NA	No
**Rijnders, 2021**	CNN; scaled GC matrix, one model per electrode combination, voting	83	87	85			0.85		Yes	No
	CNN; scaled GC matrix, FP1, F3 and P3 electrodes	80	77	78			0.79		Yes	No
**Zelig, 2021**	ROC; rate of PSWE							0.72	Yes	No
	ROC; rate of PSWE (only early (<72 h) EEG)							0.82	Yes	No
Ahmadi, 2020*	Gradient Boost; microstates-derived features			75.4	79.2	75.4			Yes	No
	SVM (Radial basis function); linear, nonlinear and connectivity, alpha			59.2	69.06	59.2			No	No
	SVM (Linear); linear, nonlinear and connectivity, beta			63.8	68.25	63.8			No	No
	RandomForest; linear, nonlinear and connectivity, delta			58.8	68.43	58.8			No	No
	SVM (Radial basis function); linear, nonlinear and connectivity, theta			53.4	64.92	53.4			No	No
	SVM (Linear); linear, nonlinear and connectivity, gamma			55.4	70.01	55.4			No	No
**Lin, 2020**	CNN; raw signal (0% overlap)	48	82	65			57.83	0.6496	No	No
	CNN; raw signal (50% overlap)	56	82	69			64.36	0.7010	No	No
	CNN; raw signal (90% overlap)	62	90	76			72.09	0.7880	No	No
	CNN; raw signal (95% overlap)	70	90	80			77.77	0.8188	No	No
**Ouyang, 2020**	XGBoost; autoregressive model errors	89.98	81.81	85.17				0.8754	Yes	No
	L1-Reg. LR; autoregressive model errors	90.47	90.47	84.83				0.8632	Yes	No
	RDA; autoregressive model errors	65.41	86.11	76				0.8908	Yes	No
**Prahbu, 2020**	MLP; KC and ApEn for 14 electrodes	95.0	98.0	96.5	98.1			0.964	Yes	No
	MLP; KC and ApEn for 6 electrodes	99	94.5	97	95.5			0.967	Yes	No
**Song, 2020**	SVM with medium Gaussian kernel; connectivity features	86.60	90.0	88.3					Yes	No
	SVM with linear kernel; connectivity features	73.3	70.0	71.70					Yes	No
	SVM fine Gaussian kernel; connectivity features	60	93.3	76.7					Yes	No
	SVM with coarse Gaussian kernel; connectivity features	96.7	43.3	70					Yes	No
**Uyttenhove, 2020**	t-VGG; raw signal	75.89	78.57	76.5		75.89			No	Yes^†^
	t-VGG GAP; raw signal	81.56	80.95	81.42		81.56			No	Yes^†^
	SVM; band power	75.18	71.43	74.32		75.18			No	Yes^†^
	RandomForest; band power	92.91	52.38	83.61		92.91			No	Yes^†^
	EEGNet; raw signal	75.89	73.81	75.41		75.89			No	Yes^†^
**Varatharajah, 2020**	Naive Bayes with Gaussian prior; band power				0.46	0.75	0.57	0.79	No	No
	SVM (radial basis function); band power				0.89	0.57	0.56	0.66	No	No
	LASSO; band power				0.89	0.56	0.55	0.76	No	No
	GNB (FT channels); band power				0.69	0.73	0.7	0.81	No	No
	SVM-RBF (FT channels); band power				0.889	0.55	0.53	0.73	No	No
	LASSO (FT channels); band power				0.38	0.5	0.43	0.82	No	No
**Yağmur, 2020**	PCA-MLP; statistical features			96	96	97			Yes	No
	LDA-MLP; statistical features			96	98	95			Yes	No
	Forward selection-MLP; statistical features			85	94	88			Yes	No
	Backward selection-MLP; statistical features			94	95	96			Yes	No
**Panwar, 2019**	ROC classifier; characteristic method analysis							0.87	Yes	Yes
**Tripathi, 2018**	Normalised band power			90					NA	No
**V, 2018**	LDA; microstates features	85.7	66.6	76.1	0.69	0.85	0.76	0.7	Yes	No
	Logistic regression; microstates features	80.9	57.1	69.0	0.65	0.8	0.72	0.67	Yes	No
**Bosl, 2017**	Linear SVM with RFE; nonlinear features (Epi vs HC+ASD)	100	100	100					Yes	No
	SVM; nonlinear features (Epi vs ASD)	72	77	75					Yes	No
**Mazzucchi, 2017**	ROC classifier, path length pre- vs. per-HV	41	100	70				0.71	Yes	Yes
**Tibdewal, 2017**	SVM; BMA-BMV	96.96	100	97.05					Yes	No
	SVM; IQR-MI	98.82	100	99.41					Yes	No
	SVM; MD-MI	98.82	100	99.41					Yes	No
	SVM; MD-IQR	97.65	100	98.82					Yes	No
**Uriguen, 2017**	ROC; spectral entropy, all channels			85				0.84	Yes	No
	ROC; spectral entropy, optimal channels	86	76	81					Yes	No
**Schmidt, 2016**	Peak alpha frequency	0	100	0^††^					No	No
	Connectivity based on PLV (mean degree)	3.3	100	10^††^					No	No
	Seizure-generating capability based on phase oscillator model	56.7	100	61.7^††^					No	No
**Dasgupta, 2015**	Ridge regression with mRMR; connectivity features			79.01				0.87	Yes	No
**Pyrzowski, 2015**	ROC, alpha score from zero-crossings analysis							0.81	Yes	No
	ROC, Shannon entropy from zero-crossings analysis							0.76	No	No
	ROC, min-entropy from zero-crossings analysis							0.71	No	No
**Rajaei, 2015**	KNN; connectivity features	85.7	100	92.8					Yes	No
**Sargolzai, 2015 (1)**	KNN; connectivity features	88.8	85.7	87.5					Yes	No
	KNN with feature selection; connectivity features			96.87					Yes	No
**Sargolzai, 2015 (2)**	GMM with PCA; connectivity features	81.8	100	88.9					Yes	No
**Schmidt, 2014**	ROC; theta band critical coupling constant	76.9	65.7		69.2			NA	Yes	No
	ROC; low-alpha band global order parameter for Fp1	71.4	74.4		NA			0.78	Yes	No
**Yang, 2014**	ANFIS; PermEn			89					Yes	No
	ANFIS; PermEn			82					Yes	No
**Sargolzaei, 2013**	KNN; connectivity features	75	100						Yes	Yes
**Cabrerizo, 2012**	ANN; linear features	96.42	95.50	96.03					Yes	No
	SVM; linear features	97.06	96.63	96.79					Yes	No
**Chaovalitwongse, 2011**	NSVM (Quadratic) dataset I; connectivity	96	100	98					No	No
	NSVM (Quadratic) dataset II; connectivity			100					No	No
	A-SFM (Euclidean), dataset I; connectivity			40					No	No
	A-SFM (T-statistics), dataset II; connectivity			0					No	No
	V-SFM (Euclidean), dataset I; connectivity			100					No	No
	V-SFM (T-statistics), dataset II; connectivity			0					No	No
**Douw, 2010**	LR; theta band SL	53	70	61					Yes	No
	LR; theta band power	58	77	NA					Yes	No
	LR; theta band SL (EEG with no IEDs)	62	76	69					Yes	No
**Luo, 2010**	ANN, top three features; linear and nonlinear features	92.2	91.7					0.883	Yes	No
	ANN, all features; linear and nonlinear features							0.908	Yes	No
**Bao, 2009**	Probabilistic NN, voting across channels for each segment, segment length 40.96 s, cut-off frequency NA, band-pass filt. NA; linear and nonlinear features	83.33	84.69	84.27					Yes	No
	Probabilistic NN, voting across channels for each segment, segment length 40.96 s, cut-off frequency 56 Hz, band-pass filt. 2–32 in 1 Hz increments; linear and non-linear features			94.07					Yes	No
**Fan, 2009**	C-SVM with gaussian kernel; connectivity			94.8					Yes	No
	C-SVM with linear kernel; connectivity			50.6					Yes	No
	SVM with gaussian kernel; connectivity			69.4					Yes	No
	SVM with linear kernel; connectivity			53.8					Yes	No
**Cassar, 2008**	ARMA model with one band (unspecified) and one electrode (unspecified)	100	65	85					Yes	No
**Poulos, 2003**	Least-squares; auto-correlation coefficient	0.83	0.90						Yes	No
**Ruseckaite, 2001**	Euclid classifier, mode of frequency spectrum (background segment)			70					No	No

* Reported from Table 3. [68]^†^Only for between test comparisons. ^††^Calculated from study. Acc: Accuracy; ANFIS: Adaptative neuro-fuzzy inference system; ANN: Artificial neural network; ARMA: Autoregressive moving average; ASD: Autism spectrum disorder; BM(A/V): Bispectrum magnitude (average/variance); CNN: Convolutional neural network; CohMean: Mean of coherence; Epi: Epilepsy; F_1_: F1-score; GC: Granger causality; GMM: Gaussian mixture model; GNB: Gaussian Naïve Bayes; HC: Healthy controls; HV: Hyperventilation; IQR: Interquartile range; KC: Kolmogorov complexity; kNN: k-nearest-neighbor; LDA: Linear discriminant analysis; LR: Logistic regression; MD: Mahalanobis distance; MI: Mutual information; MLP: Multilayer perceptron; mRMR: Maximum relevance minimum redundancy; NEAD: Non-epileptic attack disorder; PCA: Principal component analysis; PLV: Phase-locking value; Prec: Precision; PSWE: Paroxystic slow wave events; RDA: Regularized discriminant analysis; Rec: Recall; RFE: Recursive feature elimination; ROC: Receiver operating characteristic curve; Sens: Sensitivity; Spec: Specificity; SFM: Support feature machine; SVM: Support vector machine; t-VGG: tiny-VGG.

**Table 5:** Glossary for technical terms related to EEG processing and machine learning.

**Terms**	**Definitions**
Linear markers	Markers derived from linear analysis, usually extracted with time-frequency decompositions like the Fourier or wavelet transform. These methods assume independent and stationary oscillating processes. Even though the EEG signal is highly non-linear and non-stationary[138], [139], this simple representation is closely tied to the way neurologists visually inspect EEG recordings.
Non-linear markers	Markers derived from the analysis of non-linear dynamics, either summarized using higher-order features such as entropy and fractal dimensions or analyzed with dynamical models like in recurrent quantitative analysis[140].
Connectivity markers	Markers derived from the analysis of the connectivity between channels (sensor-based) or brain sources (source-based) based on a connectivity measure that represents the strength of pairwise connections between sensors or sources, respectively. Connectivity markers are higher-order features that characterize the network model.
Microstates analysis	In this approach, maps of global field power are extracted at distinct timepoints in the EEG[141]. Using a clustering algorithm, the most characteristic maps for each group are identified—the EEG microstates—on which new EEGs are back-fitted. Features are extracted from time series of microstates, including the duration and coverage (fraction of time that the microstate is active).
Independent component analysis	Blind source separation algorithm that attempts to separate the signal into statistically independent components[136]. The estimated sources are visually inspected to identify those that correspond to artifacts (e.g., blinking, heart rhythms), which are removed before reconstructing the signal with the remaining components. A machine-learning model can also be trained to automatically identify artifactual components[100].
Deep learning	Type of machine learning where models are composed of layers of nonlinear functions that progressively abstract the representation of the raw input data, enabling to capture arbitrarily complex functions[142]**.** For EEG, the main advantage of deep learning is that the model learns its own representation of the input data, without the need of preprocessing and feature extraction.
Support vector machine (SVM)	Soft margin classifier that finds the hyperplane which maximizes the distance between it and the closest observation of each class (called the support vectors). With kernels, the SVM can be optimized on non-linear feature space in a computationally efficient way.
Cross-validation (CV)	Method for validation of predictive performances of a machine-learning model. K-fold CV: in this approach, the dataset is split into K-folds. For K iterations, the machine learning algorithm is optimised on all but one folds, and its predictions are evaluated on the remaining fold. Repeated or nested-CV: the CV is either repeated with different partitions of the data or nested into a second CV loop, both leading to more robust performance estimates[97].

### 4.1 EEG recording

The range of EEG recording times was 12 s to 3 h (median: 20 min, interquartile range [IQR]: 5–25 min). The median number of electrodes was 19 (IQR: 19–20.5). In studies reporting EEG montage, 21 (58%) used a referential, and four (11%) used a bipolar montage. Sampling frequency ranged from 114 Hz to 512 Hz, with two studies using frequencies above 1000 Hz (2500 [39] and 5000 Hz [54]). The most common sampling frequencies were either 256 Hz or 250 Hz (n = 21, 58%).

### 4.2 Segmentation and handling of artifact

Thirty-six of the 37 studies (97%) segmented EEG recordings before analysis. Twenty-three studies (62%) performed manual selection of the EEG segments, most according to pre-specified criteria such as absence of artifacts or absence of ictal activity. The duration of individual EEG segments ranged between 1 and 240 s (median=11, IQR: 8–32). One study used the whole, non-segmented EEG for classification [37].

Ten studies (27%) performed artifact detection and rejection, most of which used independent component analysis (ICA, Table 3) [38], [39], [40], [47], [54], [58], [64]. Another approach was to remove outlier segments based on amplitude [54], [57]. Twenty studies (54%) identified artefactual segments visually from the recordings. No study evaluated the inter-rater reliability of manual selection nor its effect on diagnostic performances.

### 4.3 Computational biomarkers of epilepsy

The computational biomarkers extracted from the EEG signal can be broadly categorized into the following categories: linear, non-linear, connectivity, and deep learning (Table 2 and Table 3). Here, we describe in more detail which features were used in the individual studies. Estimation of the diagnostic accuracy of each individual feature, along with comparison between features, was deemed uninformative due to high risk of bias.

#### 4.3.1 Linear

The relative spectral powers of delta (≤4 Hz), theta (4–8 Hz), alpha (8–13 Hz), beta (13–40 Hz), and gamma (≥ 40 Hz) bands were used in seven studies [42], [44], [51], [52], [63], [65], [67]. Two studies compared alpha sub-bands (6–9 Hz vs. 8–13 Hz and 7.5–10.5 Hz vs. 10.5–13.5 Hz) [15], [39]. These studies used several methods to extract the power spectral density, including Fast-Fourier transform [39], [44], [51], [67] and an autoregressive model [52]. In all but two studies [15], [68], relative band power was a useful discriminant between groups. Besides estimating power spectral density, autoregressive models can be used to quantify the stationarity of a signal by computing its prediction errors [69], and autocorrelation functions provide a similar information. The linear methods for quantifying stationarity did not show consistent results across studies [18], [69], [70]. Hjorth parameters quantify higher-order statistical moments of the signal in both the time- and frequency-domains [71]. They were extracted in two studies and seemed discriminant [51], [67].

Zelig et al. (2022) extracted Paroxysmal Slow Wave Events (PSWE), defined as 2-second EEG windows with a median peak frequency of < 6 Hz. In a cohort of 70 patients presenting after a first seizure, the rate of PSWE in the first routine EEG could predict the diagnosis of epilepsy at 18-month with an AUROC of 0.72, regardless of ASM.

#### 4.3.2 Non-linear

Entropy was the most common feature explored for the automated diagnosis of epilepsy. Several algorithms have been developed to estimate entropy from finite physiological time-series. In the selected studies, Shannon [68], [70], Spectral [57], [68], Approximate [66], [70], Permutation [50], Sample (multiscale) [45], [50], Fuzzy [72], and Renyi entropy [68] were used. In some cases, entropy was computed after processing the signal in different frequency bands, either with wavelet decomposition [68] or using a coarse-graining procedure [45], allowing to estimate its value across different timescales.

Other nonlinear features included fractal dimensions (using Higuchi’s, Katz’, and Petrosian’s algorithms) [67], [68], Hurst index (or exponent) [70], zero-crossing interval analysis [56], recurrence quantitative analysis [45], characteristic response analysis (a model of the dynamics of the covariance matrix through time) [41], the bispectrum magnitude (variance and average) [72], periodicity [70], and Kolmogorov complexity [66].

#### 4.3.3 Connectivity and topographical markers

All but one [55] of the 14 connectivity studies used a sensor-based connectivity analysis [15], [42], [47], [48], [49], [53], [58], [59], [60], [62], [64], [66], [68]. The connectivity measure varied widely across studies (Table 2). A challenge of connectivity estimation is that some sensors may be spuriously connected due to a common underlying source or because of scalp conduction. When these spurious connections occur, the two sensors are phase-aligned (zero-lag), while a “true” communication between brain regions has a small time lag [73]. Therefore, one technique is to use a connectivity measure that accounts for this time lag, which four studies used: lagged correlation [59], lagged coherence [55], Granger’s causality [64], and transfer entropy [38]. Another approach reported in two studies was a model of interactions between brain regions based on the Kuramoto oscillator to calculate parameters that could embody the seizure-generating capacity of the network [15], [59]. Each study analysed the connectivity across several frequency bands.

Once the connectivity matrix is estimated for each frequency band, the studies either directly used the matrix as input into a classification scheme [60], [62], [64] or calculated higher-order features that describe the topology of the underlying network (Table 2). The discriminative power of each feature was not consistent across studies. Only network efficiency (the average of the shortest path between pairs of nodes) was higher in people without epilepsy in the three studies in which it was analyzed [38], [55], [58]. Overall, the discriminative power of the network features was highly dependent on hyperparameters [38], [47], frequency band [42], [53], [55], [64], and localization [53], [64], with conflicting results between studies. None of the studies performed statistical testing to test the robustness of the estimated network or check it against a random network [74].

Microstates analysis was reported in two studies. Although this analysis can be applied to different frequency bands independently, one study found that microstates features were only discriminant in the beta band [68].

#### 4.3.4 Deep learning

Four studies used deep learning (DL) models, specifically convolutional neural networks (CNN) [43], [63], [64], [65]**.** Two studies performed significant preprocessing on the input signal: one pre-transformed the EEG into connectivity matrices based on Granger causality (6 ×6–24 ×24 images) [64] and the other into power spectral density plots (32 ×32 images) [63]. The other two studies input the raw EEG data (18 channels x 2 s and 19 channels x 10 s, both 256 Hz), with minimal processing (band pass and notch filtering) [43], [65]. The number of layers in the CNNs ranged from one convolution layer to three blocks of two convolution layers. The number of parameters was not available, but was estimated from figures to range from ∼2 960 [64] to ∼92 000. [43].

The number of recordings used for optimization in those four studies was 48, 32, < 252, and < 1 648 (estimated from figures for the last two studies). When training curves were provided, they revealed overfitting on the training data (i.e., no decrease in loss on the validation set). No study used pre-training nor data augmentation.

Optimization algorithms included Stochastic gradient descent, Adaptive moment estimation (ADAM), and Root Mean Squared Propagation (RMSProp). Only one study used regularization (L2-regularization with dropout) [65].

#### 4.3.5 Comparison between feature extraction approaches

**Fig. 4:**
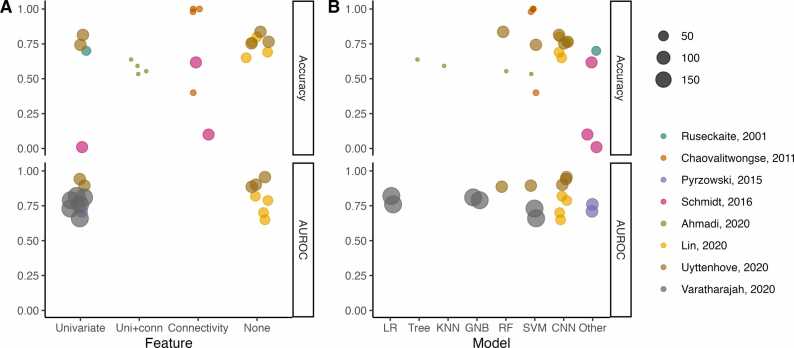
Diagnostic performance of studies with no data leakage; all studies reported either Accuracy, AUROC, or both. Each point denotes an individual test reported in the studies (some studies reporting more than one test). A: Performance as a function of the class of feature extracted from the EEG signal. B: Performance as a function of the machine learning model. The size of the points represents sample size. AUROC: Area under the receiver-operating-characteristic curve; CNN: Convolutional neural network; GNB: Gaussian Naïve Bayes; KNN: K-Nearest-neighbor; LR: Logistic regression; PSD: Power spectral density; RF: Random Forest; Uni+conn: Combination of univariate and connectivity features.

Fig. 4A depicts AUROC and accuracy for the eight studies that did not show data leakage (sharing of information between training and testing set; see Section 4.4.3). Tests based on connectivity markers showed high variability in AUROC and accuracy compared to univariate features with no feature extraction. This finding could reflect the heterogeneous data processing related to connectivity analyses. Among these eight studies, only one investigated connectivity and non-linear features across various frequency bands. [68] This study indicated a tendency for improved accuracy when using features extracted from the beta band (Katz’s fractal dimension, Shannon entropy, Spectral entropy, Renyi entropy, and microstates features). When assessing all 37 studies, the most performant band varied between the delta, [37] theta, [49], [56] alpha, [49], [52], [59] and beta [53], [68] bands.

### 4.4 Machine learning methods

Thirty of 37 studies (81%) used machine learning to map the extracted features to epilepsy diagnosis. The remaining studies used a receiver operating characteristic (ROC) curve or simple thresholding based on a single, continuous biomarker value [15], [37], [41], [55], [56], [57], [59]. Supplementary Table S1 summarizes machine learning approaches in included studies.

#### 4.4.1 Algorithms

The support vector machine (SVM) was the most popular across all studies (n = 10, 27%) [38], [39], [45], [51], [60], [62], [63], [65], [68], [72]. Studies mainly used radial basis function kernels and polynomial kernels. In some cases, the SVM was directly applied to the pairwise connectivity measures [60], [62].

Multilayer perceptrons were also widely used (n = 7, 19%) [40], [50], [51], [66], [67], [68], [70]. Four studies (11%) used convolutional neural networks (discussed in the previous section) [43], [63], [64], [65]. Regression algorithm included logistic regression (n = 6, 16%) [18], [39], [42], [54], [58], [63], and linear discriminant analysis (n = 3, 8%) [18], [52], [54], often combined with regularization to put a constraint on the value of the parameters and reduce overfitting. Other classifiers included K-nearest-neighbors (n = 5, 14%) [46], [47], [48], [53], [68], gaussian mixture models or naïve bayes with gaussian kernel [39], [49], random forest or other decision trees [65], [68], and gradient boosting [18], [68].

Six studies (16%) compared classifiers to one-another [18], [39], [51], [63], [65], [68]. In Ahmadi et al. (2020), SVM (linear and radial basis function [RBF] kernels) seemed superior to gradient boosting, decision trees, and random forest across experiments. In Varatharajah et al. (2020), both regularized logistic regression and naïve bayes had superior performances over SVM (RBF kernel). In these two studies, classifiers were trained on extracted features and not on the raw, EEG time series. Uyttenhove et al. (2020) compared CNNs trained on the preprocessed windowed EEG signal to an SVM and a random forest trained on the band powers of delta and alpha sub-bands (1.5–2 Hz, 10.5–11 Hz, 11–11.5 Hz, and 11.5–12 Hz). They showed that CNNs had higher performance when tested on the TUH Epilepsy Corpus. For each of these studies, there were few details on the hyperparameter optimization of each model, which could have significantly affected the final performances.

#### 4.4.2 Performance evaluation

The most common method for evaluating classification performances was K-fold cross-validation (CV, with K = 5 or 10), used in 10 studies (27%) [38], [43], [45], [50], [53], [54], [58], [60], [64], [66], [68]. A common variation was leave-one-out (or leave-one-pair-out) CV (n = 8, 22%) [15], [39], [52], [54], [56], [62], [67], [68]. Repeated or nested-CV was used in five studies (14%) [18], [39], [43], [51], [53]. A potential advantage of CV or repeated testing is that they evaluate the variance of the performances across different partitions of the data. However, none of the studies that performed CV or repeated testing reported the variance of the estimated performances [18], [48], [55], [65].

One common culprit for data leakage was to train the classification algorithm on epochs from one EEG recording, and then evaluate it on different epochs from the same EEG. This could be prevented by grouping together epochs from a single subject into the same data subset. This was done in eight studies (22%) [15], [39], [43], [52], [53], [62], [65], [68].

In five studies (14%), the authors evaluated performances in a dedicated testing set [18], [51], [61], [65], [72]. However, this prevented data leakage in only two of these studies (see next section) [61], [65]. For the remaining studies, performances were either tested directly on the training data or were not detailed.

#### 4.4.3 Data leakage and train-test loops

Eight studies (22%) did not present data leakage for at least one classification pipeline [15], [39], [43], [56], [61], [62], [65], [68]. In machine learning, data leakage refers to the unintentional sharing of information from the testing set to the training set, resulting in over-optimistic validation performances. Data leakage occurred at different stages of the processing pipeline: feature extraction [38], [41], [52], [54], [68], [69], [72], feature selection [15], [40], [42], [45], [46], [52], [53], [54], [55], [56], [57], [58], [63], [70], [72], and model training and evaluation [15], [37], [39], [40], [41], [42], [43], [46], [48], [49], [52], [53], [55], [59], [62], [65], [68], [69]. Fig. 4 illustrates the most common examples of data leakage. For feature extraction, data leakage occurred when the computation of features required a model to be fitted to the whole dataset, which, for these studies, included samples from the testing set (Fig. 4**B**). Feature selection caused data leakage in all studies that performed it (Fig. 4**C**) [15], [40], [42], [45], [46], [52], [53], [54], [55], [56], [57], [58], [63], [70], [72]. Eight studies (22%) reported grouping samples from the same patients in the same set (training or evaluation), avoiding data leakage that would have occurred by training on epochs from one EEG and testing on different epochs from the same EEG (Fig. 4**E**) [15], [39], [43], [52], [53], [62], [65], [68]. Ten studies (27%) did not use any external validation method when assessing diagnostic performance [37], [40], [41], [42], [46], [48], [49], [55], [59], [69].

**Fig. 5:**
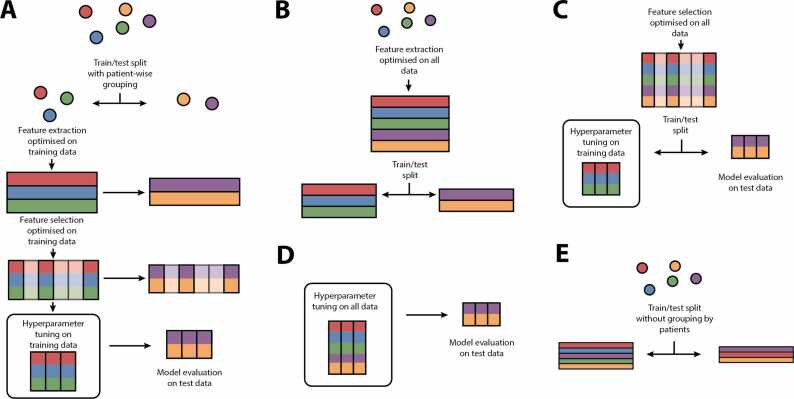
Examples of common sources of data leakage in the included studies. The circles represent individual observations (e.g., a single EEG recording) and rectangles are the feature vectors for that single observation. Elements in red, blue, and green are in the training set, and elements in purple and orange, the testing set. A: Typical machine learning pipeline without data leakage. First, the individuals (circles) are split into a training and a testing set. Then, features are extracted from the training set; the optimized feature extraction algorithm is then applied to the testing set. Third, a feature selection algorithm is applied to the training data, and the optimal features are selected on the testing data. Fourth, the machine learning hyperparameters are tuned on the training data, and the best model is evaluated on the testing set. B: Data leakage during feature extraction, where the feature extraction algorithm is optimized on both training and testing data (before the train/test split). C: Data leakage during feature selection, where the optimal features are selected on both training and testing data. D: Data leakage during model evaluation, where the hyperparameters are tuned on both training and testing data. E: Data leakage during train/test split, where samples from the same individuals (e.g., different epochs of the same EEG) are present in both training and testing data.

#### 4.4.4 Study reproducibility

Six studies (16%) were judged reproducible [18], [43], [45], [53], [57], [59]. The following elements were the most frequently unspecified or poorly specified in studies judged as not reproducible: hyperparameter tuning (n = 16, 43%), EEG segmentation (n = 16, 43%), model evaluation (n = 9, 24%), feature extraction (n = 9, 24%), and handling of artifacts (n = 9, 24%).

In addition, only three studies (8%) did not involve manual selection of EEG segments [37], [60], [62]. Two studies (5%) provided a certain access to parts of the computer code used for the analysis [59], [64]. Four studies (10%) used publicly available data [63], [64], [65], [66].

#### 4.4.5 Comparison between machine learning approaches

A comparison of the different machine learning models for the eight studies with no data leakage is shown in Fig. 4**B**. When looking at individual studies, we observed a trend towards higher performances for simpler models in two studies (logistic regression, decision trees), [39], [68] although the magnitude of this difference in accuracies was not reported.

Across all eight studies, deep learning did not clearly show higher performances. However, a direct comparison between deep learning and traditional ML was done in only one study. [65] This study used two different CNN architectures: EEGNet [75], with one split convolution layer (∼1 000 parameters) and tiny-VGG (t-VGG) [76], a compact version of the Visual Geometry Group (VGG) architecture with 3 blocks of 2 convolution layers (∼21 000 parameters) [65]. They showed that the t-VGG had superior performance for the diagnosis of epilepsy. Few details, however, were provided regarding the training hyperparameters of EEGNet in their study, while they used heavy regularization during the training of t-VGG. In another study, increasing the overlap percentage during segmentation improved performances of CNN, which may be related to the increased size of the training sample with larger overlap (6000 vs. 11,960 samples). [43] A rule-of-thumb for determining the sample size requirement of a deep neural network is to use 50 training data points per parameter. [77] In the four deep learning studies, the number of parameters were approximately 33,100, [65] 92,000, [43] 2900, [64] and 19,700 [63] (estimations based on study texts). Thus, we estimate that the number of data points represented 7.2%, [65] 0.3%, [43] 0.04% [64], and 0.004% [63] of the sample recommended sample size. [77].

## 5 Discussion

We performed a systematic review of studies reporting computational biomarkers of routine EEG to assess their diagnostic performance for epilepsy. We screened 10 166 studies and included 37 studies, the largest of which had 192 subjects. The included studies reported biomarkers used to classify epilepsy based on linear (43%), non-linear (27%), connectivity (38%), and convolutional neural network (10%) models. Although reported accuracy measures were often high (up to 100%), methodological issues such as spectrum effects and data leakage were ubiquitous and limit the interpretation of these estimates. Therefore, despite several studies published in the last 20 years, the diagnostic performance of computational analysis of routine EEG remains unclear.

The discovery of new reliable interictal markers of epilepsy from routine EEG would significantly impact the approach to the diagnosis of epilepsy [24]. While routine EEG plays an important part in the classification of epilepsy types and identification of epilepsy syndromes, its role in the diagnosis of epilepsy is mostly restricted to capturing IEDs in patients presenting after a first unprovoked seizure [8], [78]. Because of the sporadic nature of IEDs, their absence cannot rule out a diagnosis of epilepsy (sensitivity), and thus their use as diagnostic biomarkers is limited [7], [8]. In addition, because of their resemblance with other physiological sharply contoured waveforms, overreliance on IEDs can lead to the misdiagnosis of epilepsy (specificity) [11], [12]. The rate of misdiagnosis in epilepsy in the community is estimated to be around 20% [3], [4]. Erroneous diagnoses carry unnecessary and harmful consequences such as stigma, adverse effects from medication, and lifestyle or employment restrictions [10]. Alternative biomarkers could counterweight the limitations of traditional EEG interpretation, potentially accelerating the diagnosis of epilepsy while reducing the burden of over-diagnosis [5]. Several modalities have been proposed as a source of diagnostic and prognostic biomarkers for epilepsy, including neuroimaging, body fluids (blood, cerebrospinal fluid), and metabolic imaging [24]. Compared to these modalities, EEG is inexpensive, technically easy to acquire, and confers functional information with high temporal resolution [79], [80]. Moreover, great effort was put in recent years to standardize the acquisition and storage of routine EEG data [81], [82]. For these reasons, EEG is an invaluable candidate in the search of new interictal markers of seizure risk [24].

We observed a high risk of bias in all included studies. Patient selection might have inflated diagnostic performances reported in most studies especially owing to adopting a “case-control” type of study design. [83], [84] In case-control diagnostic studies, the diagnostic test aims to identify cases (patients with epilepsy) and controls (patients without epilepsy), where both groups are drawn from separate populations (e.g., patients undergoing presurgical evaluation vs. patients evaluated for headaches). Many clinical conditions affect the EEG signal, such as psychiatric diseases, brain lesions, cognitive disorders, medication, and age [8], [85], [86], [87], [88], [89]; failure to account for systematic differences in these co-morbidities between cases and controls can result in spectrum effects. This can largely inflate performances of diagnostic test accuracy studies. In this review, the impact of patient selection could not be measured because no studies showed low risk of bias in this domain. The better way to perform patient selection in diagnostic test accuracy studies is to use a consecutive sample of participants respecting common selection criteria (e.g., consecutive patients presenting to the emergency department after a first seizure) [90]. This second option tends to better replicate the scenario where the test will be applied when deployed in real-life [91]. The need for more robust patient selection methodology is echoed in other recent systematic reviews on the use of machine learning in healthcare [92], [93], [94].

Validation of the biomarkers’ performances was another important issue in the evaluation of the risk of bias. Only 22% of the studies did not exhibit data leakage during training and classification. Data leakage occurs when a sample in the evaluation set is used to optimize the classification method [95]. This can happen when the features are computed (feature extraction), when the most discriminative features are selected (feature selection), during the selection of hyperparameters (model tuning), or during the optimization of the classification algorithm (model training) (Fig. 4) [96]. Classification algorithms frequently require setting specific hyperparameters that control the flexibility of the model and its capacity to fit a particular dataset; the selection of these hyperparameters was largely unreported and can bias accuracy measures upwards [97]. Robust model selection and hyper-parameter tuning do not involve the testing data, an important principle when evaluating clinical predictive algorithms [97], [98]. The studies with low risk of bias in the Index test domain demonstrated smaller inter-test variability. This may highlight the impact of avoiding data leakage on a more precise estimation of diagnostic performance for a given population. [91] However, this estimate may not be generalizable to real-world scenarios depending on the selection criteria used for the study population.

We reported the methods used for processing the EEG signal and predicting the diagnosis, including pre-processing techniques, algorithms for feature extraction, and classification models. A widespread limitation of the EEG processing was the manual selection of artifact-free segments in 54% of studies, without quantifying the effect of this operation on downstream performances, introducing a potential source of bias. Ideally, the processing pipeline should be fully automated and identical for all patients, including artifact detection and segmentation (for example, see [99], [100]). Because of its relatively low signal-to-noise ratio, EEG data is subject to high variability induced by the recording setting, apparel, and even patient-related characteristics (e.g., hair, muscle activation, eye movements). [101], [102], [103] In future studies, large-scale initiatives integrating rEEG recordings from multiple centers along with a more widespread use of ambulatory EEG as a diagnostic tool in patients with first unprovoked seizures [104] will likely amplify this challenge. Automated methods for artifact detection and rejection based on deep neural networks are promising alternatives to manual identification, [105], [106], [107] but their capacity to increase downstream performances remains unclear. [108].

EEGs were segmented into short epochs (typically ≤1 min) in almost all studies. As a result, the longer-term dynamics of the computational markers were unexplored. The diagnosis of epilepsy relates to a chronically higher propensity to seizure, yet the markers that are evaluated operate on the millisecond-second timescale. Some models of interictal-ictal transition derived from intracranial EEG suggest that there may exist a slowly fluctuating state that embodies the seizure threshold [109], an observation replicated in studies of chronic EEG [110]. Taking these slower dynamics into account could improve the accuracy of seizure propensity assessment on routine EEG.

We could not perform a reliable comparison of the wide range of potential computational biomarkers explored in included studies. It is uncertain whether the studied biomarkers truly represent seizure propensity or are instead a proxy of other conditions that are more prevalent in people with epilepsy, such as ASM therapy and brain lesions. Several markers such as band power were highly discriminant in some studies [39], [44], [52], but not better than chance in others [15], [68]. Most studies evaluated a wide range of features over several frequency bands on a small group of patients, without assessing the variance of the results or using robust model evaluation techniques. In particular, connectivity features were impacted by a low robustness to hyperparameters, which was directly demonstrated in two of the included studies [38], [47]. Statistical validation of network models could help characterize the usefulness of connectivity analysis in future studies [111], [112]. As shown in Fig. 4, methods that take the raw EEG data as input and do not rely on feature extraction may be more robust to the variability introduced by processing parameters and potentially generalize better to external data.

The SVM was the most popular classification algorithm. In a study on the performance of several model architectures for tabular data, ensembles of decision trees (XGBoost, LightGBM, and CatBoost) significantly outperformed deep neural networks and other architectures [113]. This category of machine learning models (initially published in 2016) [114] was used in only two studies (outperforming other models in only one) [18], [68]. An ensemble of decision trees have a high complexity and, without proper hyperparameter tuning and regularization, can easily overfit small datasets, which could explain this discrepancy [114]. For smaller datasets, regularized logistic regression and SVM, which have very few hyperparameters, might be preferable. For complex input such as raw EEG signal, deep neural networks have shown promising performances for the identification and prediction of seizures [115], flagging of abnormal recordings [116], and detection of interictal discharges [11]. Only two studies used a deep convolutional neural network on the raw EEG data [43], [65]. The sample sizes of the deep learning studies were orders of magnitude smaller (between 0.004% and 7% of suggested sample size) than what is generally suggested. [77] Combined with the complexity and noise of the scalp EEG data, the sample sizes may not have been sufficient to harness the full capacity of deep neural networks. Several questions regarding deep learning remain unanswered, including the minimal quantity of EEGs required, the impact of architecture and optimizer, and the potential benefits of pretraining, self-supervised training, data augmentation, and transfer learning, all of which improved performances in other EEG-related classification tasks [117]. For seizure prediction, where the task consists in predicting (usually from long-term scalp or intracranial EEG data) when a seizure will start minutes or hours in advance, transformer models are becoming the state-of-the-art on benchmark datasets. [118], [119], [120] Transformers are typically larger and more data-hungry than CNN, but might scale better to large datasets. [121].

Understanding the predictions of a machine learning model can provide insights into the neurophysiological manifestations of epilepsy, monitor biases and flaws in the data, and improve acceptability from patients and physicians [122]. This concept is referred to as interpretability, and can take many forms. In one study, the authors used a Kuramoto model to estimate local and global seizure susceptibility from the patients’ EEGs [59]. The Kuramoto model is an abstract model of the synchronization between weakly coupled oscillators. As such, their experiment led to the hypothesis that there is a higher coupling strength in patients with generalized epilepsy compared to controls. In another study, the authors investigated the gradient flow through the fitted CNN to identify the regions in the input data that had the highest impact on the CNN’s prediction [65]. They found that the EEG regions with highest impact had highly epileptiform anomalies; this would however indicate a limited utility of this approach in the absence of IEDs. In general, interpretability is improved by imposing constraints and sparsity to a machine-learning model [123]. Constraints include imposition of structure and abstraction of unimportant features. Sparsity means that the model is described by a small number of critical parameters. For predicting the diagnosis of epilepsy, an ideal model would provide: 1) a quantification of seizure recurrence risk, 2) actionable parameters (e.g., parameters that can be modified by medication), and 3) parameters that are related to the dynamics of the cortical activity (susceptibility to bifurcations, altered connectivity, shifts in frequency). Such a model would have the potential to extrapolate to other use cases (e.g., intensive care unit, predict epileptogenicity, post-operatory outcome).

How automated analysis of EEG will integrate into the current diagnostic pathway is yet to be determined. The exact role will likely depend on whether these algorithms prove more sensitive or specific to epilepsy than the current diagnostic approach. If these algorithms were sensitive (i.e*.*, low false negative rate), they could be used as a screening test to exclude epilepsy in patients with low clinical suspicion, reducing the burden of repeat EEGs or accelerating the investigation for alternative conditions. If specific (i.e*.*, low false positive rate), they could be considered as add-ons to IEDs in patients with high pre-test probability, either to individualize the estimation of seizure recurrence risk for a single patient or to provide electrophysiological evidence of epilepsy in patients who do not show IEDs on repeat EEGs. The overhead of the automated analysis of EEG is small and these algorithms could easily be integrated into EEG interpretation software. Even large deep learning models require little computational capabilities to provide inference. [124] Although inference is cheap, training modern and robust ML models requires important computational resources and large, multicenter datasets, both of which come at a potentially very high cost. Another and even more important caveat is the risk of increasing social and racial disparities that are well documented in epilepsy. [125], [126], [127] By training on data that contain these bias, researchers must take active steps to identify and correct for these inequities. [128], [129] Simulation studies could help quantify the net clinical benefits and provide an accurate cost-benefits estimate, [130] which will ultimately hinge on the diagnostic performances of the algorithms.

The strengths of our study include the pre-registration and publication of our study protocol in a peer-reviewed journal, the inclusion of all computational methods, and rigorous study selection and data extraction processes conducted by two independent and mutually blinded reviewers. Our study, however, has limitations. We excluded studies that only used automated IEDs and seizure detection. Although such methods are reported [131], [132], any increment in accuracy from computational identification of IEDs and seizure for the diagnosis of epilepsy is intrinsically limited by their low prevalence in routine EEGs [133]. We considered reports using both IEDs/seizures and other biomarkers of epilepsy on routine EEG, but did not identify such studies. Our goal was to study biomarkers that may help circumvent known drawbacks of human expert assessment and reduce the current reliance on epileptiform discharges. Another limitation is the high methodological heterogeneity in the studies which prevented any meta-analyses to be performed, although this limitation reflects the state of the existing literature on the topic of interest.

### 5.1 Recommendations

Considering these findings, we propose the following recommendations to guide future studies of computational analysis of EEG for the diagnosis of epilepsy.

#### 5.1.1 Patient selection, reference standard, and study design

Patient selection should be carefully planned to minimize spectrum effect when assessing diagnostic performances. The test should be validated on a consecutive sample of patients that represent the population in which the index test is intended to be used. The reference standard—the diagnosis of epilepsy—should be clearly defined, applied to all patients, and be based on the ILAE’s practical definition of epilepsy [1]. Enough details should be provided in the reporting of the study to adequately assess the risk of bias of the methodology, including the start and end of the recruitment period, the number of patients screened for inclusion, the number excluded and reasons for their exclusion. Contemporary reporting standards are available to improve the planification and reporting of diagnostic accuracy studies [134]. Although great effort has been made to publicly share EEG data, current available databases do not yet satisfy these criteria.

#### 5.1.2 Validation of performances

The presence of data leakage must be evaluated at every step of the processing pipeline, from the pre-processing of the EEG signal (using methods that rely on multiple EEGs) to the selection of optimal features and the optimization of the classification algorithm, regardless of the method used for validating performances. Ideally, external validation should also be assessed on independent data, both in terms of location (e.g., different hospital) and time (non-overlapping time periods). Reporting of diagnostic accuracy should be accompanied by a measure of statistical precision, such as a 95% confidence interval.

#### 5.1.3 Code and algorithms

Code should be publicly available to ensure reproducibility of all analyses. Automated segmentation of EEG should be preferred to manual selection of EEG segments. In the case of connectivity analyses, there should be rigorous statistical validation of the network model to increase confidence in the model’s prediction. Interpretability should be at the forefront of the design of the machine learning model to increase acceptability and monitor for biases during learning. Transformers, deep CNNs, and graph neural network have revolutionized our capacity to model complex data and potentially remove the dependency on data pre-processing; they should be considered important candidates for the analysis of clinical EEG.

#### 5.1.4 Clinical translation and applicability

Future studies should provide clear paths towards clinical translation. They should more intentionally target specific clinical populations (e.g., patients evaluated after a first unprovoked seizure, patients with unexplained neurological episodes suspicious of epilepsy) and directly measure the clinical impact compared to current approaches. Small, proof-of-concept studies should make way for larger, multicenter evaluations of diagnostic performances. Integration into clinical workflow, including ease of use, time saved/lost, integration with available tools, computational requirements, and challenges in applicability, should be provided.

## 6 Conclusion

After two decades of research, the current literature provides insufficient evidence to assess the utility of computational analysis of routine EEG to diagnose epilepsy. Studies in this field are at high risk of bias, specifically for patient selection, the definition of the reference standard, and the methodology used to validate diagnostic accuracy. Because of its accessibility and information content, the routine EEG remains an important contender in the search for quantitative markers of seizure risk. We provide recommendations that could guide the design of future studies to maximize the potential for clinical translation of this technology.

## Authors’ contributions

Each author contributed to this systematic review. EL planned the study, reviewed the search strategy, participated in the data collection and analysis, drafted the initial manuscript, and is the guarantor of the review. DT, FL, DKN, and EBA participated in the conception of the study. BN and RP designed the search strategy. JNB, BR, and OG participated in data collection. JNB, BR, OG, DT, MRK, FL, DKN, and EBA provided content expertise and critically reviewed the manuscript. All authors reviewed and approved the final manuscript.

## Author agreement statement

We the undersigned declare that this manuscript is original, has not been published before and is not currently being considered for publication elsewhere. We confirm that the manuscript has been read and approved by all named authors and that there are no other persons who satisfied the criteria for authorship but are not listed. We further confirm that the order of authors listed in the manuscript has been approved by all of us.

## Declaration of Competing Interest

ÉL is supported by a scholarship from the Canadian Institute of Health Research (CIHR). BR wishes to acknowledge financial support from the Centre for Clinical Brain Sciences of the University of Edinburgh, the CIHR, the Fonds de recherche du Québec—Santé (FRQS) and the Ministère de la Santé et des Services sociaux du Québec, and the Power Corporation of Canada Chair in Neurosciences of the University of Montreal. MRK and DKN report unrestricted educational grants from UCB and Eisai, and research grants for investigator-initiated studies from UCB and Eisai. DKN and FL are supported by the Canada Research Chairs Program, the Canadian Institutes of Health Research, and Natural Sciences and Engineering Research Council of Canada. OG is supported by the Institute for Data Valorization (IVADO). EBA is supported by IVADO (51628), the CHUM research center (51616), and the Brain Canada Foundation (76097). Funding sources had no role in the design or conduct of the study.

## Data Availability

Data collected for this study will be available upon reasonable request.
