# Supplementary material for: Computer-assisted analysis of routine EEG to identify hidden biomarkers of epilepsy: A systematic review
Source: Comput Struct Biotechnol J. 2023 Dec 10;24:66–86. doi: 10.1016/j.csbj.2023.12.006 (PMC10776381; doi:10.1016/j.csbj.2023.12.006)
Supplement: Supplementary file 1 — Supplementary material. [file mmc1.docx]

# Supplementary material

| **Table S1:** Feature extraction, validation method, and data leakage for each study | | | | | | | | |
| --- | --- | --- | --- | --- | --- | --- | --- | --- |
| **Study** | **Features** | **Leakage: extraction** | **Feature selection** | **Leakage: selection** | **Classifier** | **Evaluation method** | **Leakage: evaluation** | **Metrics** |
| **Cao, 2021** | Connectivity based on MI, correlation (maximum, mean, lag), coherence (maximum, mean), wavelet coherence (maximum, mean), and PLV for each band | No | One-way ANOVA test with p < 0.00001 | Yes | K-Nearest-Neighbor | Repeated 10-fold CV with grouped subjects (5 iterations) | No | Accuracy; AUROC |
| **Guerrero, 2021** | Relative band power of frequency bands and some selected interactions (based on trial and error), frequency spectrum image (RGB) for CNN | No | Manual | Yes | Regularized LR with manually selected C parameter, ANN with 6 hidden layers and L2 regularization, SVM with (RBF) with manually selected hyperparameters, CNN with 5 layers | Unclear | Unclear | Accuracy; AUROC; Precision and recall; F1-score |
| **Rijnders, 2021** | Connectivity matrix based on Granger causality for multiple combinations of electrodes at 4 frequency band (scaled and not scaled) | No | None | - | CNN (1 convolution layer), one model for each of three electrode combinations combined with voting | 10-fold CV (one prediction per example) repeated for three different CNN, and combining predictions (voting) | Yes | Accuracy; Sensitivity and specificity; F1-score |
| **Zelig, 2021** | Paroxysmal slow wave events (rate per min) averaged over all channels | No | None | - | ROC classifier | Tested on training set | Yes | AUROC |
| **Ahmadi, 2020** | Univariate (energy, entropy [Shannon entropy, Spectral entropy, Renyi entropy], fractal dimension [fractal box dimension, Higuchi fractal dimension. Katz fractal dimension]), functional network based on horizontal visibility graph synchronization (clustering coefficient, strength, betweenness centrality, eigenvector centrality, largest eigenvalue), EEG band-specific microstates (occurrence, duration, and coverage) | Only for microstates | None | - | K-Nearest-Neighbors, Decision tree, Neural network, SVM (linear and RBF), RandomForest, Naive Bayes, Gradient boosting | Leave-one-patient-of-each-class-out CV | No | Accuracy; AUROC; Precision and recall |
| **Lin, 2020** | Raw EEG signal | - | - | - | CNN with raw signal as input | Nested CV (subject-wise split) | No | Accuracy; Sensitivity and specificity; AUROC; F1-score; Discriminative power |
| **Ouyang, 2020** | Total prediction error of autoregressive model for each channel | No | None | - | Extreme gradient boosting (XGBoost), Logistic regression (regularized), Discriminant analysis (regularized) | Bootstrapped test set (20% of data, 50 iterations), 10-fold CV on training set to tune hyperparameters | Yes | Accuracy; Sensitivity and specificity; AUROC |
| **Prahbu, 2020** | Kolmogorov complexity, Approximate entropy | No | Manual selection of best channels | Yes | Multi-layer perceptron with all channels and with a subset of channels | 10-fold CV | Yes | Accuracy; Sensitivity and specificity; AUROC; Precision and recall |
| **Song, 2020** | Average clustering coefficient and network efficiency for varying thresholds with dimensionality reduction using Tucker decomposition | Yes | None | - | SVM based on connectivity features | 10-fold CV | No | Accuracy; Sensitivity and specificity |
| **Uyttenhove, 2020** | Raw signal (CNN), Univariate features: band power for 1.5-2Hz, 10.5-11Hz, 11-11.5Hz, and 11.5-12Hz (SVM and RandomForest) | No | None | - | t-VGG CNN with 3 convolution blocks (with and without Global average pooling), SVM, RandomForest, EEGNet | Held-out testing set (grouped subjects) | No | Accuracy; Sensitivity and specificity; Precision and recall; PRC AUC |
| **Varatharajah, 2020** | Band power in low-alpha and high-alpha for eight channels | No | None | - | Naive Bayes with gaussian prior, SVM with RBF kernel, LASSO | Repeated leave-one-subject-out cross-validation (5) | No | AUROC; Precision and recall; F1-score |
| **Yağmur, 2020** | Discrete cosine transform (average, variance, standard deviation, skewness, kurtosis) for each electrode with PCA | No | TBA, DAA, forward feature selection, backward feature selection | Yes | MLP | Tested on training set | Yes | Accuracy; Precision and recall |
| **Panwar, 2019** | Characteristic response analysis | Yes | No | - | ROC classifier (Bhattacharyya distance between the observed distribution and the ideal Gaussian distribution [threshold set to 0.02]) | Tested on training set | Yes | AUROC |
| **Tripathi, 2018** | Normalized PSD for every frequency band | No | Unclear | Unclear | Unclear | Unclear | Unclear | Accuracy |
| **V, 2018** | Microstate based on global field potential maps (duration, frequency of occurrence, percentage of time coverage) | Yes | Information-based feature selection and manual selection of optimal features | Yes | LDA on two selected features, LR on two selected features | Leave-one-out CV (accuracy, sensitivity, specificity) and 10-fold CV (ROC) | No | Accuracy; Sensitivity and specificity; AUROC |
| **Bosl, 2017** | Multiscale entropy, Recurrent quantitative analysis (recurrence rate, determinism, laminarity, max line length, entropy, trapping time) | No | Recursive feature elimination | Yes | Linear SVM | 10-fold cross-validation | No | Accuracy; Sensitivity and specificity |
| **Mazzucchi, 2017** | Source-based connectivity markers (characteristic path length and clustering coefficient) based on lagged coherence | No | Manual selection of best feature | Yes | ROC classifier | Tested on training data | Yes | Accuracy; Sensitivity and specificity; AUROC; PPV and NPV |
| **Tibdewal, 2017** | Bispectrum magnitude average and variance, MI, Mahalanobis distance, Interquartile range, Fuzzy entropy | For MI only | Visual inspection for Fuzzy entropy, unclear for others | Yes | SVM with various feature pairs | Testing set (epoch-wise, not grouping by patient) | Yes | Accuracy; Sensitivity and specificity |
| **Uriguen, 2017** | Local and Shannon spectral entropy at different frequency bands | No | Permutation-based statistical testing | Yes | ROC curve using all/optimal channels | Tested on training set (all channels) and 5-fold CV (optimal channels) | Yes | Accuracy; Sensitivity and specificity; AUROC |
| **Schmidt, 2016** | Peak alpha frequency over occipital channels, connectivity based on phase-locking factor in low alpha band (mean degree), seizure-generating capability based on "local coupling constant" from phase oscillator model in low alpha band | No | None | - | Thresholding to give highest sensitivity at 100% specificity and repeated for highest specificity at 100% sensitivity | Leave-one-subject-out cross-validation | No | Sensitivity and specificity; specificity at 100% sensitivity; sensitivity at 100% specificity |
| **Dasgupta, 2015** | Connectivity based on Pearson's correlation coefficient (graph connectivity, density, energy, network clustering coefficient, network efficiency, rich club coefficient, small world index) | No | mRMR | Yes | L2-regularized LR | 5-fold CV | No | Accuracy; AUROC |
| **Pyrzowski, 2015** | Alpha score (value of intervals between zero-crossings), shannon entropy of the interval distribution, min entropy of the interval distribution, interval spectrum-derived marker:  statistical parameters (mean, mode, median, standard deviation, interquartile range) | For alpha-score only | Kruskall-Wallis test (p-value < 0.05) | Yes | ROC classifier | Leave-one-pair-out | No | AUROC |
| **Rajaei, 2015** | Connectivity based on correlation between probabilities of phase-space recurrence (link density, average degree, rich club metric, S metric, algeabric connectivity, radius) | No | None | - | K-means clustering | Testing on training set | Yes | Accuracy; Sensitivity and specificity |
| **Sargolzai, 2015 (1)** | Connectivity based on cosine similarity (link density, average degree per node (ADN), degree, avg ADN of neighboring nodes for every vertex, avg closeness centrality, avg betweenness centrality, weighted clustering coefficient, rich club metric, s-metric, algebraic connectivity, energy) | No | Sequential Feature Selection based on General Linear Model (GLM-SFS) | Yes | K-means clustering, with and without feature selection | Testing on training data and leave-one-sample-out cross-validation | Yes | Accuracy; Sensitivity and specificity; PPV and NPV |
| **Sargolzai, 2015 (2)** | Connectivity based features based on cosine similarity (link density, averaged closeness centrality, clustering coefficient, rich club metric, S-metric, algrebraic connectivity, energy) | No | None | No | Gaussian mixture-model with PCA (using individual epochs, proportion of epochs classified is used to assign final prediction) | Tested on training set | Yes | Accuracy; Sensitivity and specificity; PPV and NPV |
| **Schmidt, 2014** | Critical coupling constant for every band derived from dynamical connectivity analysis, Global order parameter for every channel derived from dynamical connectivity analysis | No | Manual selection of significant bands | Yes | ROC classifier | Tested on training set | Yes | Sensitivity and specificity; AUROC; Precision and recall; PPV |
| **Yang, 2014** | Sample entropy and permutation entropy | No | None | - | ANFIS classifier | 10-fold cross-validation (epoch wise) | Yes | Accuracy |
| **Sargolzaei, 2013** | Connectivity based on cosine similarity (link density, avg degree per node (ADN), degree, avg of ADN of neighboring nodes for every vertex, avg closeness centrality, avg centrality score, weighted clustering coefficient, rich club metric, S-metric, algebraic connectivity, graph energy, avg path length, avg vertex eccentricity, graph radius) | No | None | - | K-means clustering | Testing on the training set | Yes | Sensitivity and specificity |
| **Cabrerizo, 2012** | Hjorth parameters (activity, mobility, complexity) and spectral power density (alpha, theta, delta, beta 1, beta 2, gamma): Average, standard deviation and SNR over all electrodes | No | None | - | ANN (Three-layer perceptron), SVM with polynomial kernel | Repeated testing set (15) | Yes | Accuracy; Sensitivity and specificity |
| **Chaovalitwongse, 2011** | Univariate features (Euclidean distance, T-Statistical distance), connectivity based on Euclidean distance | No | Integrated into SFM | No | Distance averaging support feature machine (A-SFM), Voting support feature machine (V-SFM), SVM, Network-based SVM (NSVM), Decision Tree (J48) | Leave-one-patient-out cross validation | No | Accuracy; Sensitivity and specificity |
| **Douw, 2010** | Synchronization likelihood, averaged over all channels and epochs for a single patient | No | Manual | Yes | LR | Testing on training set | Yes | Accuracy; Sensitivity and specificity |
| **Luo, 2010** | Univariate features: SD of autocorrelation, Hurst indices, periodicity, approximate entropy, Shannon entropy, periodicity of smoothed signal | No | T-test | Yes | ANN with all and top three features as input | Unclear | Unclear | Sensitivity and specificity; AUROC |
| **Bao, 2009** | Power spectrum relative intensity ratio, Fractal dimension (Petrosian, Higuchi), Hjorth parameters (complexity and mobility) | No | None | - | Probabilistic neural network | Leave-one-sample-out CV | Yes | Accuracy |
| **Fan, 2009** | Connectivity measure based on Euclidean distance | No | None | - | SVM (linear and gaussian kernels), C-SVM (linear and gaussian kernels) | 5-fold CV | Yes | Accuracy |
| **Cassar, 2008** | ARMA-based power spectral density | Yes | T-test | Yes | LDA | Leave-one-subject-out CV | No | Accuracy; Sensitivity and specificity |
| **Poulos, 2003** | Auto-correlation coefficient | Yes | None | - | Least squares method | Testing on training set | Yes | Sensitivity and specificity |
| **Ruseckaite, 2001** | Mode of frequency spectrum | No | None | - | “Euclid classifier” | Held out testing set | No | Accuracy |
| ANFIS: Adaptative neuro-fuzzy inference system; ANN: Artificial neural network; ARMA: Autoregressive moving average; ASD: Autism spectrum disorder; BM(A/V): Bispectrum magnitude (average/variance); CNN: Convolutional neural network; CohMean: Mean of coherence; Epi: Epilepsy; F_1_: F1-score; GC: Granger causality; GMM: Gaussian mixture model; GNB: Gaussian Naïve Bayes; HC: Healthy controls; HV: Hyperventilation; IQR: Interquartile range; KC: Kolmogorov complexity; kNN: k-nearest-neighbor; LDA: Linear discriminant analysis; LR: Logistic regression; MD: Mahalanobis distance; MI: Mutual information; MLP: Multilayer perceptron; mRMR: Maximum relevance minimum redundancy; NEAD: Non-epileptic attack disorder; PCA: Principal component analysis; PLV: Phase-locking value; Prec: Precision; PSWE: Paroxystic slow wave events; RDA: Regularized discriminant analysis; Rec: Recall; RFE: Recursive feature elimination; ROC: Receiver operating characteristic curve; Sens: Sensitivity; Spec: Specificity; SFM: Support feature machine; SVM: Support vector machine; t-VGG: tiny-VGG. | | | | | | | | |

# Figure S1: Risk of bias by individual studies


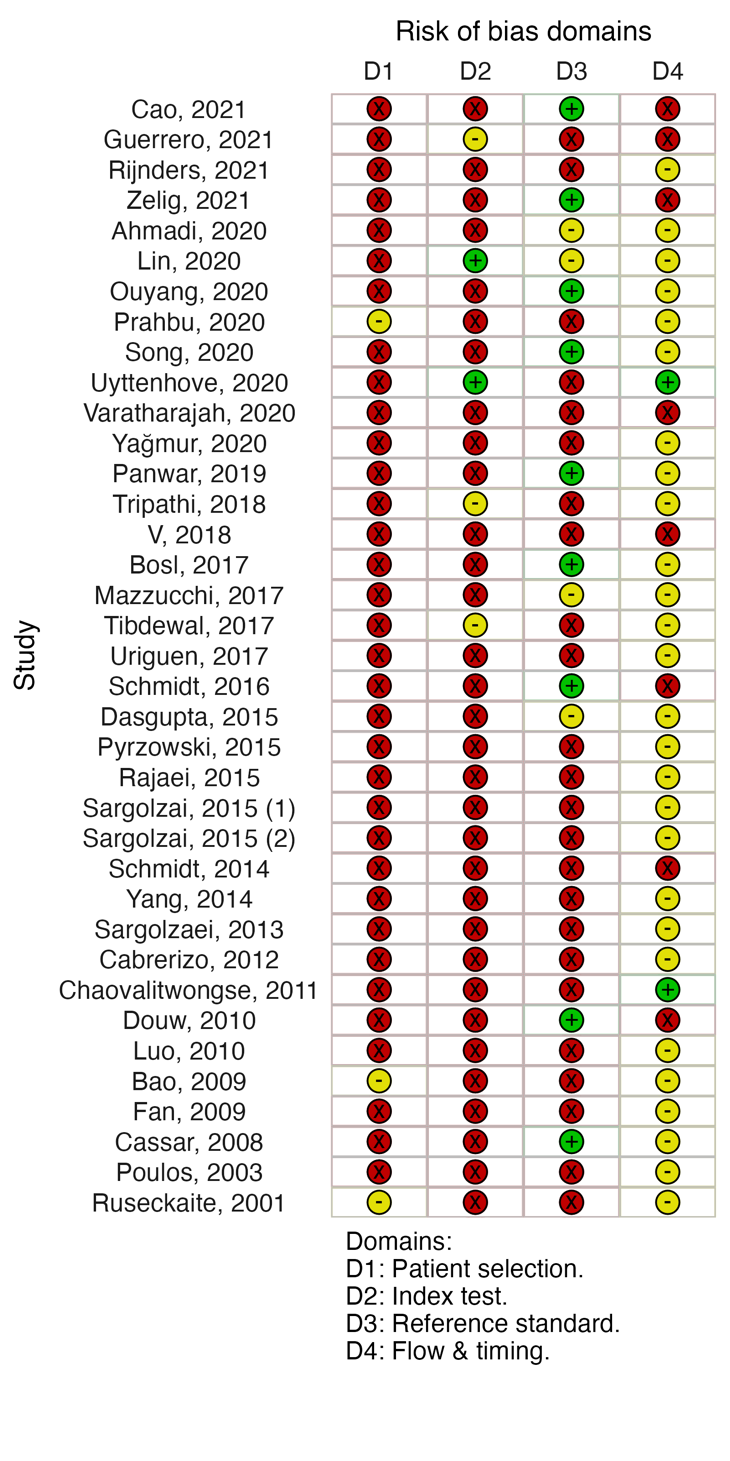


**Figure S1:** Risk of bias of individual studies across the four PRISMA domains using a modified QUADAS-2 scale. Green circles indicate a low risk of bias, red circles indicate a high risk of bias, and yellow circle, an unclear risk of bias.

# Figure S2: Diagnostic performances and risk of bias


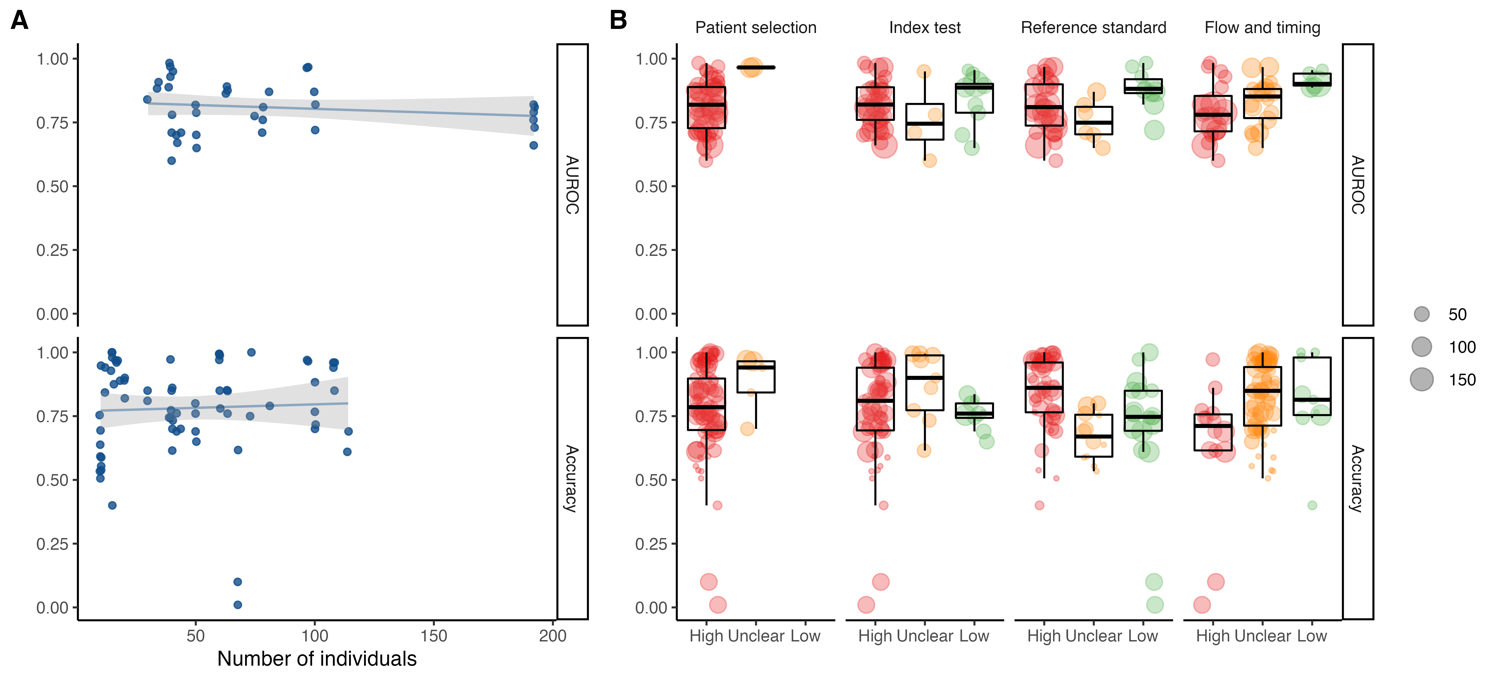


**Figure S2:** Correlation of sample size and risk of bias with diagnostic performance, with each point denoting an individual test reported in the studies (some studies reporting more than one test). **A**: Sample size and reported diagnostic performance metrics (AUROC and accuracy). **B**: Influence of risk of bias in each of the four QUADAS-2 domains on reported diagnostic performance (see **Table S2** for detailed evaluation of each study).

## Table S2: Detailed consensus on the risk of bias for individual studies

Signaling questions for the evaluation of risk-of-bias:^1^

- **Patient selection**: a. Is the population representative of clinical practice? b. Are inclusion and exclusion criteria identical for cases (patients with epilepsy) and controls? c. Are withdrawals explained and appropriate? If individual EEG segments were excluded, were the same criteria used for all segments?
- **Index test:** a. Were the protocols used for recording the EEG identical in all patients, irrespective of the epilepsy diagnosis? b. Was the index test validated on an independent sample of patients (patients which were not used to identify the index test’s threshold or train the learning algorithm)?
- **Reference standard**: a. Are the criteria used for the diagnosis of epilepsy specified and acceptable (likely to correctly classify the target condition)? b. Was the reference standard assessment independent and blinded to the index test?
- **Flow and timing**: a. Did the whole sample undergo the reference standard? b. Did the whole sample undergo the same reference standard? c. Was the time lapse between reference standard and EEG acceptable? d. Was the same data used in the index method available at the time of the reference standard? e. Were all EEGs included in the analysis?

| **Study** | **Patient selection** | **Index test** | **Reference standard** | **Flow and timing** |
| --- | --- | --- | --- | --- |
| **Cao, 2021** | a. No: cases and controls drawn from distinct databases.  b. No: different criteria for cases and controls.  c. Yes: no exclusion.  Decision:^†^ **High RoB** | a. No: recording protocols were different for cases and controls.  b. No: samples from testing set used for feature selection.  Decision: **High RoB** | a. Yes  b. Yes  Decision: **Low RoB** | a. No: controls did not undergo any reference standard.  b. No.  c. Not specified.  d. Yes.  e. Number of eligible EEGs not specified.  Decision: **High RoB** |
| **Guerrero, 2021** | a. No.  b. Inclusion and exclusion criteria are not specified.  c. No: some patients were excluded without justification.  Decision: **High RoB** | a. Recording protocols are not specified.  b. Not specified.  Decision: **Unclear RoB** | a. No: epilepsy diagnosis is based on the index EEG.  b. No.  Decision: **High RoB** | a. Unclear*  b. Unclear*  c. Unclear*  d. Unclear*  e. No: some EEGs were excluded without justification.  Decision: **Unclear RoB** |
| **Rijnders, 2021** | a. No.  b. No: different criteria for cases and controls.  c. Not all withdrawals are clearly explained.  Decision: **High RoB** | a. Yes.  b. No: samples from testing set used for feature selection and hyperparameter optimization.  Decision: **High RoB** | a. No: epilepsy diagnosis is based on the index EEG.  b. No.  Decision: **High RoB** | a. Unclear*  b. Unclear*  c. Unclear*  d. Unclear*  e. No: some EEGs were excluded without justification.  Decision: **Unclear RoB** |
| **Zelig, 2021** | a. No: some patients without diagnostic uncertainty are included as controls.  b. No: different criteria for cases and controls.  c. No withdrawals.  Decision: **High RoB** | a. Yes.  b. No: no independent set was used for validation.  Decision: **High RoB** | a. Yes  b. Yes  Decision: **Low RoB** | a. Yes.  b. No: separate reference standard used for a subset of the controls.  c. Yes.  d. Yes.  e. Yes.  Decision: **High RoB** |
| **Ahmadi, 2020** | a. No.  b. Inclusion and exclusion criteria are not clearly specified.  c. Yes: no exclusion.  Decision: **High RoB** | a. Not specified.  b. No: samples from testing set used to generate features.  Decision: **High RoB** | a. No: Criteria for reference standard not specified.  b. NA  Decision: **Unclear RoB** | a. Unclear*  b. Unclear*  c. Unclear*  d. Unclear*  e. Yes.  Decision: **Unclear RoB** |
| **Lin, 2020** | a. No.  b. Inclusion and exclusion criteria are not clearly specified.  c. Yes: no exclusion.  Decision: **High RoB** | a. Yes  b. Yes  Decision: **Low RoB** | a. Not enough details provided.  b. Not enough details provided.  Decision: **Unclear RoB** | a. Unclear*  b. Unclear*  c. Unclear*  d. Unclear*  e. Yes.  Decision: **Unclear RoB** |
| **Ouyang, 2020** | a. No.  b. No: different criteria for cases and controls.  c. No withdrawals.  Decision: **High RoB** | a. Yes.  b. No: train/test split not stratified by individual.  Decision: **High RoB** | a. Yes  b. Yes  Decision: **Low RoB** | a. Unclear*  b. Unclear*  c. Unclear*  d. Unclear*  e. Yes.  Decision: **Unclear RoB** |
| **Prahbu, 2020** | a. Inclusion criteria are not clearly defined.  b. Not enough details provided.  c. No withdrawals.  Decision: **Unclear RoB** | a. Yes.  b. No: samples from testing set used for hyperparameter optimization.  Decision: **High RoB** | a. No: Criteria for reference standard not specified.  b. NA  Decision: **High RoB** | a. Unclear*  b. Unclear*  c. Unclear*  d. Unclear*  e. Yes.  Decision: **Unclear RoB** |
| **Song, 2020** | a. Inclusion criteria are not clearly defined.  b. No: different criteria for cases and controls.  c. No withdrawals.  Decision: **High RoB** | a. Yes.  b. No: samples from testing set used for hyperparameter optimization.  Decision: **High RoB** | a. No: Criteria for reference standard not specified.  b. NA  Decision: **High RoB** | a. Unclear*  b. Unclear*  c. Unclear*  d. Unclear*  e. Yes.  Decision: **Unclear RoB** |
| **Uyttenhove, 2020** | a. Yes.  b. No: different criteria for cases and controls.  c. No withdrawals.  Decision: **High RoB** | a. Yes.  b. Yes.  Decision: **Low RoB** | a. No: epilepsy diagnosis is based on the index EEG.  b. No.  Decision: **High RoB** | a. Yes.  b. Yes.  c. Yes.  d. Yes.  e. Yes.  Decision: **Unclear RoB** |
| **Varatharajah, 2020** | a. No: patients with DRFE vs. healthy controls.  b. No: different criteria for cases and controls.  c. No: some patients were withdrawn for EEG artifacts after a non-blinded review of their EEG.  Decision: **High RoB** | a. No: EEG recording protocols are different for cases and controls.  b. Yes.  Decision: **High RoB** | a. No: epilepsy diagnosis is based on the index EEG.  b. No.  Decision: **High RoB** | a. No: different data sources for cases and controls.  b. No.  c. Unclear*  d. Unclear*  e. Some withdrawals were not explained.  Decision: **High RoB** |
| **Yağmur, 2020** | a. Inclusion criteria are not clearly defined.  b. No: different criteria for cases and controls.  c. No withdrawals.  Decision: **High RoB** | a. Not specified.  b. No: no independent set was used for validation.  Decision: **High RoB** | a. No: epilepsy diagnosis is based on the index EEG.  b. No.  Decision: **High RoB** | a. Unclear*  b. Unclear*  c. Unclear*  d. Unclear*  e. Yes.  Decision: **Unclear RoB** |
| **Panwar, 2019** | a. Inclusion criteria are not clearly defined.  b. No: different criteria for cases and controls.  c. No withdrawals.  Decision: **High RoB** | a. Not specified.  b. No: samples from testing set used to set decision threshold of the model.  Decision: **High RoB** | a. Yes  b. Yes  Decision: **Low RoB** | a. Yes.  b. Yes.  c. Time lapse is not specified.  d. Yes.  e. Yes.  Decision: **Unclear RoB** |
| **Tripathi, 2018** | a. Inclusion criteria are not clearly defined.  b. No: different criteria for cases and controls.  c. No withdrawals.  Decision: **High RoB** | a. Recording protocols are not specified.  b. Not enough details provided.  Decision: **Unclear RoB** | a. No: Criteria for reference standard not clearly specified.  b. NA  Decision: **High RoB** | a. Unclear*  b. Unclear*  c. Unclear*  d. Unclear*  e. Yes.  Decision: **Unclear RoB** |
| **V, 2018** | a. No: only males, patients with DRFE vs. healthy controls.  b. No: different criteria for cases and controls.  c. Yes.  Decision: **High RoB** | a. Not specified.  b. No: no independent set was used for validation.  Decision: **High RoB** | a. No: Criteria for reference standard not clearly specified.  b. NA  Decision: **High RoB** | a. Unclear*  b. Unclear*  c. Unclear*  d. Unclear*  e. Yes.  Decision: **Unclear RoB** |
| **Bosl, 2017** | a. Inclusion criteria are not clearly defined.  b. No: different criteria for cases and controls.  c. Criteria for segment selection are not clearly specified.  Decision: **High RoB** | a. Yes  b. No: samples from testing set used for feature selection.  Decision: **High RoB** | a. Yes  b. Yes  Decision: **Low RoB** | a. Yes  b. Yes  c. Time lapse is not specified.  d. Yes  e. Yes  Decision: **Unclear RoB** |
| **Mazzucchi, 2017** | a. No.  b. No: different criteria for cases and controls.  c. No withdrawals.  Decision: **High RoB** | a. Yes  b. No: no independent set was used for validation.  Decision: **High RoB** | a. No: Criteria for reference standard not clearly specified.  b. NA  Decision: **High RoB** | a. Unclear*  b. Unclear*  c. Unclear*  d. Unclear*  e. Yes.  Decision: **Unclear RoB** |
| **Tibdewal, 2017** | a. No: presurgical evaluation vs. healthy controls.  b. No: different criteria for cases and controls.  c. No withdrawals.  Decision: **High RoB** | a. Recording protocols are not specified.  b. Not enough details provided.  Decision: **Unclear RoB** | a. No: Criteria for reference standard not clearly specified.  b. NA  Decision: **High RoB** | a. Unclear*  b. Unclear*  c. Unclear*  d. Unclear*  e. Yes.  Decision: **Unclear RoB** |
| **Uriguen, 2017** | a. No: patients with IGE vs. healthy controls, different age distributions.  b. No: different criteria for cases and controls.  c. No withdrawals.  Decision: **High RoB** | a. Yes  b. No: no independent set was used for validation.  Decision: **High RoB** | a. No: Criteria for reference standard not clearly specified.  b. NA  Decision: **High RoB** | a. Unclear*  b. Unclear*  c. Unclear*  d. Unclear*  e. Yes.  Decision: **Unclear RoB** |
| **Schmidt, 2016** | a. Yes.  b. No: different criteria for cases and controls.  c. No withdrawals.  Decision: **High RoB** | a. No: EEGs are recorded at different institutions for cases and controls.  b. Yes.  Decision: **High RoB** | a. Yes  b. Yes  Decision: **Low RoB** | a. No  b. No  c. Not specified.  d. Yes.  e. Yes.  Decision: **High RoB** |
| **Dasgupta, 2015** | a. No.  b. No: different criteria for cases and controls.  c. No withdrawals.  Decision: **High RoB** | a. Yes  b. No: samples from testing set used for feature selection.  Decision: **High RoB** | a. No: Criteria for reference standard not specified.  b. NA  Decision: **High RoB** | a. Unclear*  b. Unclear*  c. Unclear*  d. Unclear*  e. Yes.  Decision: **Unclear RoB** |
| **Pyrzowski, 2015** | a. No.  b. No: different criteria for cases and controls.  c. No withdrawals.  Decision: **High RoB** | a. Yes  b. No: samples from testing set used for feature selection.  Decision: **High RoB** | a. No: Criteria for reference standard not specified.  b. NA  Decision: **High RoB** | a. Unclear*  b. Unclear*  c. Unclear*  d. Unclear*  e. Yes.  Decision: **Unclear RoB** |
| **Rajaei, 2015** | a. No.  b. No: different criteria for cases and controls.  c. Unclear: one EEG had 4 segments but all others, 10.  Decision: **High RoB** | a. Not specified.  b. No: no independent set was used for validation.  Decision: **High RoB** | a. No: Criteria for reference standard not specified.  b. NA.  Decision: **High RoB** | a. Unclear*  b. Unclear*  c. Unclear*  d. Unclear*  e. Unclear: one EEG had 4 segments but all others, 10.  Decision: **Unclear RoB** |
| **Sargolzai, 2015 (1)** | a. No.  b. No: different criteria for cases and controls.  c. No withdrawals.  Decision: **High RoB** | a. Yes  b. No: no independent set was used for validation.  Decision: **High RoB** | a. No: Criteria for reference standard not specified.  b. NA.  Decision: **High RoB** | a. Unclear*  b. Unclear*  c. Unclear*  d. Unclear*  e. Unclear: one EEG had 4 segments but all others, 10.  Decision: **Unclear RoB** |
| **Sargolzai, 2015 (2)** | a. No.  b. No: different criteria for cases and controls.  c. No withdrawals.  Decision: **High RoB** | a. Yes  b. No: samples from testing set used for feature selection and no independent set was used for validation.  Decision: **High RoB** | a. No: Criteria for reference standard not specified.  b. NA.  Decision: **High RoB** | a. Unclear*  b. Unclear*  c. Unclear*  d. Unclear*  e. Yes.  Decision: **Unclear RoB** |
| **Schmidt, 2014** | a. No: cases are only patients with IGE.  b. No: different criteria for cases and controls.  c. No withdrawals.  Decision: **High RoB** | a. Yes  b. No: no independent set was used for validation.  Decision: **High RoB** | a. No: Criteria for reference standard not specified.  b. NA.  Decision: **High RoB** | a. No.  b. No.  c. Unclear*  d. Unclear*  e. Yes.  Decision: **High RoB** |
| **Yang, 2014** | a. No: patients with EE vs. healthy children.  b. No: different criteria for cases and controls.  c. No withdrawals.  Decision: **High RoB** | a. Yes.  b. No: train/test split not stratified by individual.  Decision: **High RoB** | a. No: Criteria for reference standard not specified.  b. NA.  Decision: **High RoB** | a. Unclear*  b. Unclear*  c. Unclear*  d. Unclear*  e. Yes.  Decision: **Unclear RoB** |
| **Sargolzaei, 2013** | a. Inclusion criteria are not clearly defined.  b. No: different criteria for cases and controls.  c. No withdrawals.  Decision: **High RoB** | a. Not specified.  b. No: no independent set was used for validation.  Decision: **High RoB** | a. No: Criteria for reference standard not specified.  b. NA.  Decision: **High RoB** | a. Unclear*  b. Unclear*  c. Unclear*  d. Unclear*  e. Yes.  Decision: **Unclear RoB** |
| **Cabrerizo, 2012** | a. Yes.  b. No: different criteria for cases and controls.  c. Yes.  Decision: **High RoB** | a. Yes.  b. No: train/test split not stratified by individual; for SVM, samples from testing set used for hyperparameters optimization.  Decision: **High RoB** | a. No: Criteria for reference standard not specified.  b. Not specified.  Decision: **High RoB** | a. Unclear*  b. Unclear*  c. Unclear*  d. Unclear*  e. Yes.  Decision: **Unclear RoB** |
| **Chaovalitwongse, 2011** | a. Patient selection not clearly defined.  b. No: different criteria for cases and controls.  c. No withdrawals  Decision: **High RoB** | a. No: different recording protocols for cases and controls  b. Yes.  Decision: **High RoB** | a. No: epilepsy diagnosis is based on the index EEG.  b. No.  Decision: **High RoB** | a. Yes.  b. Yes.  c. Yes.  d. Yes.  e. Yes.  Decision: **Low RoB** |
| **Douw, 2010** | a. Yes: patients presenting after a first unprovoked seizure.  b. Yes.  c. No: some patients were withdrawn for EEG artifacts after a non-blinded review of their EEG.  Decision: **High RoB** | a. Yes  b. No: no independent set was used for validation.  Decision: **High RoB** | a. Yes  b. Yes  Decision: **Low RoB** | a. Yes  b. Yes  c. Yes.  d. Yes  e. No: some eligible EEGs were excluded.  Decision: **High RoB** |
| **Luo, 2010** | a. Patient selection not clearly specified.  b. No: different criteria for cases and controls.  c. No withdrawals  Decision: **High RoB** | a. Recording protocol not specified.  b. No: samples from testing set used for feature selection.  Decision: **High RoB** | a. No: Criteria for reference standard not specified.  b. NA  Decision: **High RoB** | a. Unclear*  b. Unclear*  c. Unclear*  d. Unclear*  e. Yes.  Decision: **Unclear RoB** |
| **Bao, 2009** | a. Population not specified.  b. Inclusion and exclusion criteria not specified.  c. Yes: no exclusion  Decision: **Unclear RoB** | a. Recording protocol not specified.  b. No: train/test split not stratified by individual.  Decision: **High RoB** | a. No: Criteria for reference standard not specified.  b. NA  Decision: **High RoB** | a. Unclear*  b. Unclear*  c. Unclear*  d. Unclear*  e. Yes.  Decision: **Unclear RoB** |
| **Fan, 2009** | a. No.  b. No: different criteria for cases and controls.  c. No withdrawals.  Decision: **High RoB** | a. Not specified.  b. No: train/test split not stratified by individual.  Decision: **High RoB** | a. No: Criteria for reference standard not specified.  b. NA  Decision: **High RoB** | a. Unclear*  b. Unclear*  c. Unclear*  d. Unclear*  e. Yes.  Decision: **Unclear RoB** |
| **Cassar, 2008** | a. No: epilepsy patients are on ASM with no active seizures.  b. No: different criteria for cases and controls.  c. Criteria for segment selection not clearly specified.  Decision: **High RoB** | a. Yes.  b. No: samples from testing set used for hyperparameters optimization.  Decision: **High RoB** | a. Yes  b. Yes  Decision: **Low RoB** | a. Yes  b. Yes  c. Time lapse is not specified.  d. Yes  e. Yes  Decision: **Unclear RoB** |
| **Poulos, 2003** | a. No.  b. No: different criteria for cases and controls.  c. No withdrawals.  Decision: **High RoB** | a. Yes  b. No: no independent set was used for validation.  Decision: **High RoB** | a. No: Criteria for reference standard not specified.  b. NA  Decision: **High RoB** | a. Unclear*  b. Unclear*  c. Unclear*  d. Unclear*  e. Yes.  Decision: **Unclear RoB** |
| **Ruseckaite, 2001** | a. Population not specified.  b. Inclusion and exclusion criteria not specified.  c. No withdrawals.  Decision: **Unclear RoB** | a. Yes  b. No: no independent set was used for validation.  Decision: **High RoB** | a. No: Criteria for reference standard not specified.  b. NA  Decision: **High RoB** | a. Unclear*  b. Unclear*  c. Unclear*  d. Unclear*  e. Yes.  Decision: **Unclear RoB** |
| ^†^Decision is “Low” if all answers are “Yes”, “Unclear” if all answers are “Yes” or “Unclear”, and “High” if any of the answer is “No”. *Criteria for reference standard not specified. ASM: antiseizure medication; DRFE: Drug-resistant focal epilepsy; EE: Epileptic encephalopathy; NA: Not applicable; RoB: Risk of Bias; SVM: Support vector machine. | | | | |

## References

1. Lemoine, É. *et al.* Computer-assisted analysis of routine electroencephalogram to identify hidden biomarkers of epilepsy: protocol for a systematic review. *BMJ Open* **13**, e066932 (2023).
