# Supplementary material for: Computer-assisted analysis of routine EEG to identify hidden biomarkers of epilepsy: A systematic review
Source: Comput Struct Biotechnol J. 2023 Dec 10;24:66–86. doi: 10.1016/j.csbj.2023.12.006 (PMC10776381; doi:10.1016/j.csbj.2023.12.006)
Supplement: Supplementary file 2 — Supplementary material. [file mmc2.pdf]

Appendix 1: Search strategy

Medline [OVID]

Ovid MEDLINE(R) and Epub Ahead of Print, In-Process, In-Data-Review & Other Non-Indexed Citations, Daily and Versions(R) <1946 to December 13, 2021>

#	Searches	Results
1	exp Electroencephalography/	173584
2	(EEG* or Electroencephalograph* or "electr* encephalograph*" or "brain wave*").tw,kf.	111352
3	1 or 2	201652
4	exp Epilepsy/	118716
5	Epilep*.tw,kf.	152323
6	(seizure* or convulsion* or infantile spasm*).tw,kf.	147989
7	(BCECTS or BECTS).tw,kf.	346
8	(panayiotopoulos adj2 syndrome*).tw,kf.	166
9	((Nodding or dravet or doose or may white or fukhura) adj2 (disease* or syndrome*)).tw,kf.	1407
10	(myoencephalopathy ragged red fiber* disease* or MERRF).tw,kf.	530
11	((Lafora or Unverricht or Landau-Kleffner or Lennox Gastaut) adj2 (disease* or syndrome* or disorder* or seizure*)).tw,kf.	2534
12	or/4-11	244612
13	exp Algorithms/	375058
14	Machine learning.tw,kf.	54804
15	((Deep or hierarchical) adj1 learning).tw,kf.	25347
16	((transfer* or representation* or network*) adj2 learning).tw,kf.	7945
17	((artificial or machine or computer or computational) adj2 intelligence).tw,kf.	19275
18	algorithm*.tw,kf.	299232
19	((data or binary or multiclass or multilabel) adj2 classification).tw,kf.	4758

20	((artificial or computational or computer* or convolutional or connectionist or mathematical) adj2 neur* network*).tw,kf.	28375
21	exp Pattern Recognition, Automated/	26085
22	(Automat* adj2 pattern* adj2 recognition*).tw,kf.	155
23	(Back* propagation* or backpropagation*).tw,kf.	4397
24	exp Bayes Theorem/	40554
25	(Bayes* adj2 (theorem or learning or analysis or approach* or forecast* or method* or prediction*)).tw,kf.	21469
26	(feature* adj2 (detecti* or extracti* or learning* or ranking* or selection*)).tw,kf.	21577
27	(Fuzzy or neurofuzzy).tw,kf.	13240
28	exp Markov chains/	15485
29	(Markov adj2 (model* or chain$1 or process*)).tw,kf.	21918
30	K nearest neighbor*.tw,kf.	3529
31	(Kernel$1 adj2 (method* or algorithm* or approach or correlation or estim* or regression or model* or string or tree)).tw,kf.	3950
32	exp Knowledge discovery/	130
33	(Knowledge adj2 discover*).tw,kf.	1589
34	exp Multifactor Dimensionality Reduction/	226
35	Dimensionality reduction*.tw,kf.	3836
36	(predicti* adj2 model*).tw,kf.	79862
37	connectom*.tw,kf.	4980
38	neur* decod*.tw,kf.	361
39	(outlier* adj2 detection*).tw,kf.	893
40	Neural networks, computer/	35265
41	(neural adj2 network*).tw,kf.	70371
42	perceptron*.tw,kf.	3390
43	radial basis function*.tw,kf.	2359
44	random forest*.tw,kf.	13717

45	recursive feature* elimination*.tw,kf.	688
46	recursive partition*.tw,kf.	2380
47	exp Support Vector Machine/	8553
48	(vector* adj2 (machine* or classifi* or network* or regression)).tw,kf.	22248
49	support vector*.tw,kf.	21483
50	rough set*.tw,kf.	397
51	((automat* or electron* or comput* or information or analytic*) adj2 (processing or reasoning)).tw,kf.	38719
52	(quantitative adj2 analys*).tw,kf.	90324
53	(Peak* adj2 (alpha* or frequenc*)).tw,kf.	5453
54	Entrop*.tw,kf.	45494
55	Lyapunov exponent*.tw,kf.	2179
56	Hjorth*.tw,kf.	184
57	Sub-band energ*.tw,kf.	18
58	exp fourier Analysis/	17272
59	(Fourier* or (cyclic adj2 (analys* or series or transform* or approach*)) or FFT).tw,kf.	87439
60	(Hilbert* adj2 transform*).tw,kf.	1008
61	(dimension* adj2 (fractal* or correlation*)).tw,kf.	8106
62	(Hurst adj2 exponent*).tw,kf.	575
63	exp wavelet analysis/	2541
64	(Wavelet* adj2 (analysis or processing or transform*)).tw,kf.	7248
65	phase locking value*.tw,kf.	311
66	Fisher information*.tw,kf.	870
67	Dynamic network*.tw,kf.	1839
68	Principal component* analys*.tw,kf.	47819
69	Independant component* analys*.tw,kf.	2
70	Functional connectivit*.tw,kf.	22171

71	(gradient* boost* or Adaboost*).tw,kf.	3337
72	(QEEG or Quantitative Electroencephalogra*).tw,kf.	1750
73	(chaotic feature* or chaos).tw,kf.	9755
74	comput*.tw,kf.	958508
75	quantitative.tw,kf.	689806
76	or/13-75	2378446
77	(sensitiv* or diagnos* or predict*).mp. or scor*.tw. or observ*.mp.	11325259
78	di.fs.	2760821
79	or/77-78	11325259
80	3 and 12 and 76 and 79	5990
81	(Animals/ or Models, animal/ or Disease models, animal/) not Humans/	4900078
82	((animal or animals or canine* or cat or cats or dog or dogs or feline or hamster* or lamb or lambs or mice or monkey or monkeys or mouse or murine or pig or pigs or piglet* or porcine or primate* or rabbit* or rats or rat or rodent* or sheep* or veterinar*) not (human* or patient* or women or men)).tw,kf.	3315730
83	81 or 82	5542727
84	80 not 83	5627
85	limit 84 to yr="1961 -Current"	5627

EMBASE [OVID]

Embase <1974 to 2021 December 13>

#	Searches	Results
1	exp electroencephalography/	124495
2	(EEG* or Electroencephalograph* or "electr* encephalograph*" or "brain wave*").tw,kf.	146325
3	1 or 2	206929
4	exp epilepsy/	251058
5	Epilep*.tw,kf.	214171
6	(seizure* or convulsion* or infantile spasm*).tw,kf.	216888
7	(BCECTS or BECTS).tw,kf.	509

8	(panayiotopoulos adj2 syndrome*).tw,kf.	249
9	((Nodding or dravet or doose or may white or fukhura) adj2 (disease* or syndrome*)).tw,kf.	2324
10	(myoencephalopathy ragged red fiber* disease* or MERRF).tw,kf.	711
11	((Lafora or Unverricht or Landau-Kleffner or Lennox Gastaut) adj2 (disease* or syndrome* or disorder* or seizure*)).tw,kf.	3984
12	or/4-11	371364
13	Machine learning/	49774
14	Machine learning.tw,kf.	63858
15	((Deep or hierarchical) adj1 learning).tw,kf.	28566
16	exp network learning/	886
17	((transfer* or representation* or network*) adj2 learning).tw,kf.	8790
18	exp artificial intelligence/	55153
19	((artificial or machine or computer or computational) adj2 intelligence).tw,kf.	23056
20	exp algorithm/	465121
21	algorithm*.tw,kf.	381089
22	((data or binary or multiclass or multilabel) adj2 classification).tw,kf.	6087
23	exp artificial neural network/	62826
24	((artificial or computational or computer* or convolutional or connectionist or mathematical) adj2 neur* network*).tw,kf.	33889
25	exp pattern recognition/ or exp automated pattern recognition/	68427
26	(Automat* adj2 pattern* adj2 recognition*).tw,kf.	199
27	exp back propagation/	2553
28	(Back* propagation* or backpropagation*).tw,kf.	5107
29	exp Bayesian learning/	4303
30	(Bayes* adj2 (theorem or learning or analysis or approach* or forecast* or method* or prediction*)).tw,kf.	24116
31	exp Feature detection/ or exp feature extraction/ or exp feature learning/ or exp feature ranking/ or exp feature selection/	31030

32	((feature* or representation) adj2 (detecti* or extracti* or learning* or ranking* or selection*)).tw,kf.	28097
33	exp fuzzy system/	4077
34	(fuzzy or neurofuzzy).tw,kf.	16138
35	exp Markov chain/ or exp Markov state model/	12093
36	(Markov adj2 (model* or chain$1 or process*)).tw,kf.	29000
37	exp k nearest neighbor/	4553
38	K nearest neighbor*.tw,kf.	4260
39	kernel method/	6720
40	(Kernel$1 adj2 (method* or algorithm* or approach or correlation or estim* or regression or model* or string or tree)).tw,kf.	4389
41	exp Knowledge discovery/	727
42	(Knowledge adj2 discover*).tw,kf.	1804
43	exp multifactor dimensionality reduction/	864
44	Dimension* reduction*.tw,kf.	7086
45	(predicti* adj2 model*).tw,kf.	105404
46	connectom*.tw,kf.	6225
47	neur* decod*.tw,kf.	433
48	exp Outlier detection/	470
49	(outlier* adj2 detection*).tw,kf.	1010
50	exp artificial neural network/	62826
51	exp Perceptron/	2478
52	perceptron*.tw,kf.	3962
53	(neural adj2 network*).tw,kf.	84786
54	exp radial basis function/	942
55	radial bas* function*.tw,kf.	2927
56	exp random forest/	14358
57	(random adj2 forest*).tw,kf.	17752

58	exp recursive feature elimination/	393
59	recursive feature* elimination*.tw,kf.	860
60	exp recursive partitioning/	462
61	recursive partition*.tw,kf.	3567
62	exp relevance vector machine/ or exp support vector machine/	28522
63	(vector* adj2 (machine* or classifi* or network* or regression)).tw,kf.	27021
64	support vector*.tw,kf.	26266
65	exp rough set/	248
66	rough set*.tw,kf.	531
67	exp online analytical processing/	187
68	((automat* or electron* or comput* or information or analytic*) adj2 (processing or reasoning)).tw,kf.	44254
69	Quantitative analysis/	367570
70	(quantitative adj2 analys*).tw,kf.	113093
71	(Peak* adj2 (alpha* or frequenc*)).tw,kf.	6315
72	Entrop*.tw,kf.	43483
73	Lyapunov exponent*.tw,kf.	1600
74	Hjorth*.tw,kf.	264
75	Sub-band energ*.tw,kf.	23
76	exp Fourier analysis/	10056
77	(Fourier* or (cyclic adj2 (analys* or series or transform* or approach*)) or FFT).tw,kf.	89584
78	Hilbert transform/	183
79	(Hilbert* adj2 transform*).tw,kf.	1253
80	(dimension* adj2 (fractal* or correlation*)).tw,kf.	8947
81	(Hurst adj2 exponent*).tw,kf.	555
82	exp wavelet transform/	2217
83	(Wavelet* adj2 (analysis or processing or transform*)).tw,kf.	9182

84	phase locking value*.tw,kf.	425
85	Fisher information*.tw,kf.	746
86	Dynamic network*.tw,kf.	1972
87	Principal component* analys*.tw,kf.	58526
88	Independent component* analys*.tw,kf.	7493
89	Functional connectivity/	21903
90	Functional connectivit*.tw,kf.	30389
91	(gradient* boost* or Adaboost*).tw,kf.	4097
92	(QEEG or Quantitative Electroencephalogra*).tw,kf.	2861
93	(chaotic feature* or chaos).tw,kf.	8412
94	comput*.tw,kf.	1156500
95	quantitative.tw,kf.	852081
96	or/13-95	2994032
97	(sensitiv* or diagnos* or predict*).mp. or scor*.tw. or observ*.mp.	14413096
98	di.fs.	3343316
99	or/97-98	14413096
100	3 and 12 and 96 and 99	8362
101	(exp animal/ or animal experiment/ or nonhuman/) not (exp human/ or human experiment/)	6801969
102	(animal or animals or canine* or dog or dogs or feline or hamster* or lamb or lambs or mice or monkey ormonkeys or mouse or murine or pig or pigs or piglet* or porcine or primate* or rabbit* or rats or rat or rodent* or sheep* or veterinar*).ti,kw,dq,jx. not (human* or patient*).mp.	2062187
103	101 or 102	6872024
104	100 not 103	7906
105	limit 104 to yr="1961 -Current"	7890
106	limit 105 to embase	5134

EBM Reviews [OVID]

All EBM Reviews - Cochrane DSR, ACP Journal Club, DARE, CCA, CCTR, CMR, HTA, and NHSEED <executed on December 14>

#	Searches	Results
1	(EEG* or Electroencephalograph* or "electr* encephalograph*" or "brain wave*").tw,kw,sh.	12245
2	Epilep*.tw,kw,sh.	10099
3	(seizure* or convulsion* or infantile spasm*).tw,kw,sh.	11675
4	(BCECTS or BECTS).tw,kw,sh.	31
5	(panayiotopoulos adj2 syndrome*).tw,kw,sh.	5
6	((Nodding or dravet or doose or may white or fukhura) adj2 (disease* or syndrome*)).tw,kw,sh.	413
7	(myoencephalopathy ragged red fiber* disease* or MERRF).tw,kw,sh.	5
8	((Lafora or Unverricht or Landau-Kleffner or Lennox Gastaut) adj2 (disease* or syndrome* or disorder* or seizure*)).tw,kw,sh.	339
9	or/2-8	16595
10	algorithm*.tw,kw.	16401
11	Machine learning.tw,kw,sh.	1918
12	((Deep or hierarchical) adj1 learning).tw,kw,sh.	708
13	((transfer* or representation* or network*) adj2 learning).tw,kw,sh.	691
14	((artificial or machine or computer or computational) adj2 intelligence).tw,kw,sh.	827
15	algorithm*.tw,kw,sh.	18549
16	((data or binary or multiclass or multilabel) adj2 classification).tw,kw,sh.	335
17	((artificial or computational or computer* or connectionist or convolutional or mathematical) adj2 neur* network*).tw,kw,sh.	782
18	(Automat* adj2 pattern* adj2 recognition*).tw,kw,sh.	15
19	(Back* propagation* or backpropagation*).tw,kw,sh.	66
20	(Bayes* adj2 (theorem or learning or analysis or approach* or forecast* or method* or prediction*)).tw,kw,sh.	1841
21	(feature* adj2 (detecti* or extracti* or learning* or ranking* or selection*)).tw,kw,sh.	607

22	(fuzzy or neurofuzzy).tw,kw,sh.	197
23	(Markov adj2 (model* or chain$1 or process*)).tw,kw,sh.	4373
24	K nearest neighbor*.tw,kw,sh.	73
25	(Kernel$1 adj2 (method* or algorithm* or approach or correlation or estim* or regression or model* or string or tree)).tw,kw,sh.	90
26	(Knowledge adj2 discover*).tw,kw,sh.	26
27	Dimensionality reduction*.tw,kw,sh.	73
28	(predicti* adj2 model*).tw,kw,sh.	5378
29	connectom*.tw,kw,sh.	308
30	neur* decod*.tw,kw,sh.	2
31	(outlier* adj2 detection*).tw,kw,sh.	14
32	perceptron*.tw,kw,sh.	76
33	(neural adj2 network*).tw,kw,sh.	1672
34	radial basis function*.tw,kw,sh.	39
35	random forest*.tw,kw,sh.	615
36	recursive feature* elimination*.tw,kw,sh.	30
37	recursive partition*.tw,kw,sh.	282
38	(vector* adj2 (machine* or classifi* or network* or regression)).tw,kw,sh.	555
39	support vector*.tw,kw,sh.	544
40	rough set*.tw,kw,sh.	3
41	((automat* or electron* or comput* or information or analytic*) adj2 (processing or reasoning)).tw,kw,sh.	7510
42	(quantitative adj2 analys*).tw,kw,sh.	8960
43	(Peak* adj2 (alpha* or frequenc*)).tw,kw,sh.	357
44	Entrop*.tw,kw,sh.	951
45	Lyapunov exponent*.tw,kw,sh.	37
46	Hjorth*.tw,kw,sh.	29
47	Sub-band energ*.tw,kw,sh.	0

48	(Fourier* or (cyclic adj2 (analys* or series or transform* or approach*)) or FFT).tw,kw,sh.	1043
49	(Hilbert* adj2 transform*).tw,kw,sh.	19
50	(dimension* adj2 (fractal* or correlation*)).tw,kw,sh.	184
51	(Hurst adj2 exponent*).tw,kw,sh.	14
52	(Wavelet* adj2 (analysis or processing or transform*)).tw,kw,sh.	126
53	phase locking value*.tw,kw,sh.	11
54	Fisher information*.tw,kw,sh.	7
55	Dynamic network*.tw,kw,sh.	12
56	Principal component* analys*.tw,kw,sh.	1207
57	Independant component* analys*.tw,kw,sh.	0
58	Functional connectivit*.tw,kw,sh.	2220
59	(gradient* boost* or Adaboost*).tw,kw,sh.	168
60	(QEEG or Quantitative Electroencephalogra*).tw,kw,sh.	448
61	(chaotic feature* or chaos).tw,kw,sh.	141
62	comput*.tw,kw,sh.	80820
63	quantitative.tw,kw,sh.	33706
64	or/10-63	145496
65	(sensitiv* or diagnos* or predict*).mp. or scor*.tw. or observ*.mp.	810011
66	di.tw,kw,sh.	17162
67	65 or 66	811399
68	1 and 9 and 64 and 67	350
69	((animal or animals or canine* or cat or cats or dog or dogs or feline or hamster* or lamb or lambs or mice or monkey or monkeys or mouse or murine or pig or pigs or piglet* or porcine or primate* or rabbit* or rats or rat or rodent* or sheep* or veterinar*) not (human* or patient* or women or men)).tw,kw,sh.	5147
70	68 not 69	346
71	limit 70 to yr="1961 -Current" [Limit not valid in DARE; records were retained]	321
72	remove duplicates from 71	315

IEEE Xplore

<executed on December 14>

((((((((All Metadata:predicted OR All Metadata:prediction OR All Metadata:predictions OR All Metadata:predicting OR All Metadata:predictive OR All Metadata:predictor OR All Metadata:predictors OR All Metadata:predicts OR All Metadata:predictability OR All Metadata:predictable OR All Metadata:predictably OR All Metadata:predictively OR All Metadata:predictiveness))) OR ((All Metadata:sensitivity OR All Metadata:sensitively OR All Metadata:sensitiveness OR All Metadata:sensitive OR All Metadata:sensitivities))) OR ((All Metadata:diagnose OR All Metadata:diagnosis OR All Metadata:diagnosed OR All Metadata:diagnoses OR All Metadata:diagnostic OR All Metadata:diagnosing OR All Metadata:diagnosable OR All Metadata:diagnostics OR All Metadata:diagnoseable OR All Metadata:diagnostical OR All Metadata:diagnostician OR All Metadata:diagnosticians OR All Metadata:diagnostically))) AND ((No Keywords Specified))) AND ((No Keywords Specified))) AND ((Index Terms:EEG) OR (Index Terms:Electroencephalograph*) OR (Index Terms: "electr* encephalograph*") OR (Index Terms: "brain wave") OR (Index Terms:"brain waves"))) OR ((Document Title:EEG) OR (Document Title:Electroencephalograph*) OR (Document Title:"electr* encephalograph*") OR (Document Title:"brain wave") OR (Document Title:"brain waves"))) AND ((Index Terms:epilep*) OR (Document Title:seizure OR Document Title:seizures OR Document Title:convulsion OR Document Title:convulsions OR Document Title:"infantile spasm" OR Document Title:"infantile spasms"))	2492

Google Scholar (using Publish or Perish)

<executed on December 21>

Electroencephalogram epilepsy [title], machine learning algorithm* diagnos* [keywords]	32 selected articles out of 32
Electroencephalography epilepsy [title], machine learning algorithm* diagnos* [keywords]	21 selected article out of 21
EEG epilepsy [title], machine learning algorithm* diagnos* [keywords]	433 sur 433

Grey literature

Alberta: Health evidence reviews

https://www.alberta.ca/health-evidence-reviews.aspx

Electroencephalography	0 selected articles out of 1
EEG	0 selected articles out of 3

Canadian Agency for Drug and Technologies in Health

https://www.cadth.ca/search?keywords

Electroencephalography	0 selected articles out of 1
EEG	0 selected articles out of 4

Health Quality Council of Alberta

https://hqca.ca/studies-and-reviews/

Electroencephalography	0 selected articles out of 0
EEG	0 selected articles out of 0

Health Quality Ontario: Health Technology Assessment

Quality Standards - Health Quality Ontario (HQO) (hqontario.ca)

Electroencephalography	1 selected article out of 7
EEG	1 selected article out of 5

INESS

https://www.inesss.qc.ca/en/publications/publications.html?tx_solr%5Bq%5D=EEG

électroencéphalographie	0 selected articles out of 5
EEG	0 selected articles out of 0

McGill University Health Centre (MUHC). Technology Assessment Unit Reports

https://muhc.ca/tau/page/tau-reports

Electroencephalography	0 selected article out of 0
EEG	0 selected articles out of 3

Newfoundland & Labrador Centre For Applied Health Research

http://www.nlcahr.mun.ca/CHRSP/CompletedCHRSP.php

Electroencephalography AND epilepsy	0 selected articles out of 37
Electroencephalogram AND epilepsy	0 selected articles out of 34
EEG AND epilepsy	0 selected articles out of 28

The Ottawa Hospital Research institute: Knowledge Synthesis Group

http://www.ohri.ca/ksgroup/

Electroencephalography	0 selected articles out of 0
Electroencephalogram	0 selected articles out of 0
EEG AND epilepsy	0 selected articles out of 7

Programs for Assessment of Technology in Health

https://www.path-hta.com/research-1

Electroencephalography	0 selected articles out of 0
Electroencephalogram	0 selected articles out of 0
EEG	0 selected articles out of 0

The International Network of Agencies for Health Technology Assessment

Publications - INAHTA

Electroencephalography	0 selected articles out of 1
Electroencephalogram	0 selected articles out of 4
EEG	0 selected articles out of 4

Horizon Scanning

Horizon Scanning - Australia and New Zealand Horizon Scanning Network - Technologies Assessed

Electroencephalography	0 selected articles out of 1
Electroencephalogram	0 selected articles out of 0
EEG	0 selected articles out of 0

Austrian Academy of Sciences

https://www.oeaw.ac.at/en/

Electroencephalography	0 selected articles out of 0
Electroencephalogram	0 selected articles out of 0
EEG	0 selected articles out of 2

Austrian Institute Of Health Technology Assessment

Welcome to Repository of AIHTA GmbH - Repository of AIHTA GmbH (lbg.ac.at)

Electroencephalography	0 selected articles out of 4
Electroencephalogram	0 selected articles out of 0

EEG	0 selected articles out of 2

KCE: Belgian health Knowledge Center

All reports - KCE (fgov.be)

Electroencephalography	0 selected articles out of 1
Electroencephalogram	0 selected articles out of 0
EEG	0 selected articles out of 1
électroencéphalographie	0 selected article out of 1

CEDIT, the Hospital-Based HTA Agency Of AP-HP

Recommendations and Reports | Cedit (aphp.fr)

Electroencephalography	0 selected articles out of 0
Electroencephalogram	0 selected articles out of 0
EEG	0 selected articles out of 1
électroencéphalographie	0 selected article out of 0

Haute Autorité de Santé

Haute Autorité de Santé - Résultat de recherche (has-sante.fr)

EEG	1 selected article out of 218
électroencéphalographie	0 selected article out of 27

Health Information and Quality Autority

Health Technology Assessments | HIQA

Electroencephalography	0 selected articles out of 0
Electroencephalogram	0 selected articles out of 0
EEG	0 selected articles out of 0

Irish Health Repository

Lenus the Irish Health Repository

Title: Electroencephalography AND epilepsy	1 selected article out of 51
Electroencephalogram	0 selected articles out of 3
Title: EEG AND epilepsy	0 selected articles out of 51

Norwegian Institute of Public Health

Norwegian Institute of Public Health - NIPH (fhi.no)

Electroencephalography	0 selected articles out of 0
Electroencephalogram	0 selected articles out of 0
EEG	0 selected articles out of 3

Swedish Agency for Health Technology Assessment And Assessment Of Social Services Home (sbu.se)

Electroencephalography	0 selected articles out of 2
Electroencephalogram	0 selected articles out of 2
EEG	0 selected articles out of 4

Healthcare Improvement Scotland

Healthcare Improvement Scotland

Electroencephalography	0 selected articles out of 0
Electroencephalogram	0 selected articles out of 0
EEG	0 selected articles out of 0

National Institute for Health and Care Excellence

NICE | The National Institute for Health and Care Excellence

electroencephalography AND epilepsy	0 selected articles out of 2
Electroencephalogram AND epilepsy	1 selected article out of 5
EEG	0 selected articles out of 9

NIHR Innovation Observatory

Innovation Observatory | Next generation search tools for the next generation. (nihr.ac.uk)

Electroencephalography	1 selected article out of 2
Electroencephalogram	0 selected articles out of 1
EEG	0 selected articles out of 5

National institute for health Research

Research Programmes (nihr.ac.uk)

electroencephalography AND epilepsy	1 selected article out of 67
Electroencephalogram AND epilepsy	0 selected articles out of 67
EEG	0 selected articles out of 67

Agency for Healthcare Research and Quality : Technology Assessment Program

Technology Assessment Program | Agency for Healthcare Research and Quality (ahrq.gov)

Electroencephalography AND epilepsy AND diagnosis	0 selected articles out of 1
Electroencephalogram AND epilepsy AND diagnosis	0 selected articles out of 78
EEG AND epilepsy AND diagnosis	0 selected articles out of 83

Agency for Healthcare Research and Quality : Evidence-Based Reports

Search Evidence-Based Reports | Agency for Healthcare Research and Quality (ahrq.gov)

Electroencephalography	0 selected articles out of 0
Electroencephalogram	0 selected articles out of 0
EEG AND epilepsy	0 selected articles out of 4

Google

intitle: Electroencephalography AND epilepsy AND machine learning AND diagnosis	3 selected articles out of 9
intitle: Electroencephalogram AND epilepsy AND machine learning AND diagnosis	0 selected articles out of 9
intitle: EEG AND epilepsy AND machine learning AND diagnosis	1 selected articles out of 9
intitle: Electroencephalography AND epilepsy AND algorithm AND diagnosis	0 selected articles out of 9
intitle: Electroencephalogram AND epilepsy AND algorithm AND diagnosis	0 selected articles out of 9
intitle: EEG AND epilepsy AND algorithm AND diagnosis	0 selected articles out of 9
